# The Prognostic Role of Right Ventricular Stroke Work Index during Liver Transplantation

**DOI:** 10.3390/jcm10174022

**Published:** 2021-09-06

**Authors:** Young Hyun Jeong, Seong-Mi Yang, Hyeyeon Cho, Jae-Woo Ju, Hwan Suk Jang, Ho-Jin Lee, Won Ho Kim

**Affiliations:** Department of Anesthesiology and Pain Medicine, Seoul National University Hospital, Seoul National University College of Medicine, #101 Daehak-ro, Jongno-gu, Seoul 03080, Korea; yhhh1130@snu.ac.kr (Y.H.J.); seongmi.yang@gmail.com (S.-M.Y.); bdbd7799@gmail.com (H.C.); jujw701@naver.com (J.-W.J.); luminsishs@snu.ac.kr (H.S.J.); zenerdiode03@gmail.com (H.-J.L.)

**Keywords:** right ventricular stroke work index, hemodynamics, liver transplantation, mortality

## Abstract

Right heart-associated hemodynamic parameters including intraoperative pulmonary vascular resistance (PVR) were reported to be associated with patient survival after liver transplantation. We investigated whether intraoperative stroke work indexes of both ventricles could have a better prognostic value than PVR. We reviewed 683 cases at a tertiary care academic medical center. We collected intraoperative variables of baseline central venous pressure, baseline right ventricle end-diastolic volume, mixed venous oxygen saturation, intraoperative PVR and right and left ventricular stroke work indexes. Time-weighted means or area under the curve of intraoperative right and left ventricular stroke work indexes were calculated as exposure variables. One-year all-cause mortality or graft failure was our primary outcome. Cox proportional hazard regression analysis was performed to evaluate the association between exposure variables and one-year all-cause mortality or graft failure. Kaplan–Meier survival curve analysis of our primary outcome was performed for different time-weighted mean ventricular stroke work index groups. Cubic spline curve analysis was performed to evaluate the linear relationship between our exposure variables and primary outcome. Time-weighted mean right ventricular stroke work index was significantly associated with one-year all-cause mortality or graft failure (hazard ratio 1.21, 95% confidence interval (CI) 1.12–1.36, *p* < 0.001). However, there was no significant association between time-weighted mean left ventricular stroke work index, time-weighted mean PVR, PVR at the end of surgery and one-year mortality. Area under the curve of right ventricular stroke work index was also significantly associated with one-year mortality or graft failure (hazard ratio 1.24, 95% CI 1.15–1.37, *p* < 0.001). Kaplan–Meier survival curve analysis showed a significant difference in the survival between different mean right ventricular stroke work index groups (Log-rank test: *p* = 0.002). Cubic spline function curve showed the gradual increase in the risk of mortality with a positive slope with time-weighted mean right ventricular stroke work index. In conclusion, intraoperative elevated right ventricular stroke work index was significantly associated with poor patient or graft survival after liver transplantation. Intraoperative right ventricular stroke work index could be an intraoperative hemodynamic goal and prognostic marker for mortality after liver transplantation.

## 1. Introduction

The hemodynamic variables during surgery are reported to be associated with mortality and graft survival after liver transplantation [1,2,3,4]. Among the directly measured and secondary calculated hemodynamic variables, variables associated with right ventricular function including pre- and intraoperative mean pulmonary artery pressure (MPAP) or pulmonary vascular resistance (PVR) were reported to be associated with posttransplant morbidity and mortality up to 36 months [5,6]. Portopulmonary hypertension is an important predictor of survival of patients with liver cirrhosis [7]. Increased PVR at the end of surgery is associated with poor clinical outcomes [5]. Additionally, right ventricular end-diastolic volume is regarded as a more sensitive index of systemic preload than central venous pressure (CVP) [8,9]. Increased baseline CVP, baseline right ventricle end-diastolic volume and mixed venous oxygen saturation (Svo_2_) during anhepatic phase are associated with increased risk of acute kidney injury after liver transplantation [2].

Right ventricular stroke work index (RVSWI) is calculated as the product of right ventricular stroke volume index and mean pulmonary arterial pressure. Left ventricular stroke work index is measured as the product of left ventricular cardiac index and mean systemic arterial pressure. RVSWI is the amount of work done by right ventricle, which is increased by pulmonary hypertension and increased PVR. Stroke volume of right heart is determined by the preload and contractility of right ventricle, in addition to afterload, the PVR. Therefore, RVSWI is a comprehensive assessment of right ventricle-associated hemodynamics and could be a prognostic factor predicting posttransplant patient and graft survival. Furthermore, intraoperative left ventricular stroke work index may have similar values of left ventricle but has never been studied as a prognostic predictor of liver transplantation.

Therefore, in this retrospective observational study, we attempted to evaluate the prognostic value of both ventricular stroke work indexes to predict postoperative patient mortality and graft survival after liver transplantation. We also sought to compare the prognostic value of these variables with the previously known right ventricle-associated hemodynamic factors including intraoperative PVR, baseline CVP, baseline right ventricle end-diastolic volume and Svo_2_ during anhepatic phase.

## 2. Materials and Methods

### 2.1. Study Design

This was a retrospective cohort study at a tertiary care academic medical center. Our study was approved by the Seoul National University Hospital Institutional Review Board (1904-118-1028, Seoul, Korea) on 26 April 2019. We reviewed the electronic medical records of 1032 consecutive adult patients who underwent elective living donor liver transplantation between 2004 and 2015. The need for written informed consent was waived by the institutional review board given the study’s retrospective design. We excluded the patients with missing pulmonary arterial pressure or right ventricular stroke volume index values, and therefore, RVSWI could not be calculated or those without left ventricular stroke volume index and, therefore, left ventricular stroke work index could not be calculated. We also excluded those with preoperative hepatorenal syndrome, because acute kidney injury was our secondary outcome.

### 2.2. Anesthesia and Hemodynamic Management

We inserted a pulmonary artery catheter routinely through a 9 Fr Advanced Venous Access catheter (Edward Lifesciences, Irvine, CA, USA) placed in the right internal jugular vein except for the patients whose internal jugular vein was already cannulated by a hemodialysis catheter. We monitored continuous cardiac output and right heart-associated parameters with the Vigilance II monitor (Edward Lifesciences, Irvine, CA, USA). To treat intraoperative hypotension, we administered crystalloid, used bolus doses of ephedrine or phenylephrine and continuously infused dopamine and/or norepinephrine and/or epinephrine according to the monitored cardiac output, Svo_2_ and systemic vascular resistance (SVR). If MPAP was greater than 35 mmHg, nitroglycerine and/or milrinone was continuously infused.

### 2.3. Data Collection

Demographic or perioperative variables known to be related to postoperative graft survival or patient mortality were collected (Table 1) [2,3,5,10,11,12]. We retrieved the study data from a prospectively collected our institutional liver transplantation database [13], which included all consecutive patients undergoing liver transplantation. All consecutive patients of our database who met our inclusion criteria during the study period were included. The date and cause of death of all patients including those lost to follow-up were collected from the Korean national statistics service (http://kostat.go.kr/portal/eng, accessed on 23 October 2019).

Intraoperative hemodynamic variables were collected which included mean arterial pressure, cardiac index, CVP, right ventricle end-diastolic volume, right ventricular stroke volume index, mean pulmonary arterial pressure, SVR and PVR [1,2]. Ventricular stroke work indexes were calculated according to the following equation [14] using component variables measured at eight times during surgery: after anesthesia induction (Time 1), 1 h after anesthesia induction (Time 2), 10 min after the beginning of the anhepatic phase (Time 3), 5 min before (Time 4) and after graft reperfusion (Time 5), 20 min after reperfusion (Time 6), 5 min after the completion of biliary reconstruction (Time 7) and at the end of surgery (Time 8). These eight time-points selected when a time-point can be used as a baseline or when it designates a surgical phase changes or when the hemodynamic instabilities are expected at that time-point according to our experiences during liver transplantation.

Right ventricular stroke work index (g·m^−2^·beat^−1^) (normal reference value: 5–10 g·m^−2^·beat^−1^).

$$\begin{array}{c} \mathrm{Right}\;\mathrm{ventricule}\;\mathrm{stroke}\;\mathrm{volume}\;\mathrm{index}\;\left(\mathrm{mL}\cdot {\mathrm{beat}}^{-1}\cdot m^{-2}\;\right)\times [\mathrm{mean}\;\mathrm{pulmonary}\;\mathrm{arterial}\;\mathrm{pressure}\;\left(\mathrm{mmHg}\right)\\ -\mathrm{central}\;\mathrm{venous}\;\mathrm{pressure}\;\left(\mathrm{mmHg}\right)]\times 0.0136 \end{array}$$

Left ventricular stroke work index (g·m^−2^·beat^−1^) (normal reference value: 45–60 g·m^−2^·beat^−1^).

$$\begin{array}{c} \mathrm{Left}\;\mathrm{ventricle}\;\mathrm{stroke}\;\mathrm{volume}\;\mathrm{index}\;\left(\mathrm{mL}\cdot {\mathrm{beat}}^{-1}\cdot m^{-2}\right)\times [\mathrm{mean}\;\mathrm{arterial}\;\mathrm{pressure}\;\left(\mathrm{mmHg}\right)\\ -\mathrm{pulmonary}\;\mathrm{artery}\;\mathrm{occlusion}\;\mathrm{pressure}\;\left(\mathrm{mmHg}\right)]\times 0.0136 \end{array}$$

Time-weighted mean right and left ventricular stroke work indexes were calculated according to the following equation:

$$\mathrm{Time}-\mathrm{weighted}\;\mathrm{mean}=\frac{\left[\left(X_{1}+X_{2}\right)\left(T_{2}-T_{1}\right)+\left(X_{2}+X_{3}\right)\left(T_{3}-T_{2}\right)+\cdots +\left(X_{n-1}+X_{n}\right)\left(T_{n}-T_{n-1}\right)\right]}{2\times \left(T_{n}-T_{1}\right)}$$

(*T_n_*, time of measurement of *X_n_*; *X_n_* = right or left ventricular stroke work index at *T_n_*).

Additionally, to evaluate the time–dose response of these variables, areas under the curve of both ventricular stroke work indexes were calculated for each patient. Area under the curve was calculated as described previously [15].

The primary outcome was one-year all-cause mortality or graft failure requiring retransplantation. Secondary postoperative clinical outcomes included postoperative hospital length of stay and ICU length of stay, in-hospital mortality, postoperative acute kidney injury defined by kidney disease and improving global outcomes criteria using serum creatinine during seven days postoperative [16].

### 2.4. Statistical Analysis

The sample size was based on the available data from all patients who underwent liver transplantation at our institution from 2004 and 2015. No statistical power calculation was performed prior to the study. STATA/MP version 15.1 (StataCorp, College Station, TX, USA) was used for Cox regression analysis, and cubic spline function curve analysis. Medcalc Statistical Software version 18.6 (MedCalc Software bvba, Ostend, Belgium) was used for survival curve analysis. SPSS software version 25.0 (IBM Corp., Armonk, NY, USA) was used for the remaining statistical analyses. Our statistical analysis plan was written and filed with a private entity of our institutional review board before data were accessed (Supplemental Text S1) [17]. *p* < 0.05 was regarded statistically significant. Categorical variables were reported as number (percentage), and either chi-square test or Fisher’s exact test was used to compare between groups depending on their expected counts. The normality of the data was determined by the Shapiro–Wilk test. Continuous variables were presented as mean ± SD for normally distributed data or median (25th, 75th percentiles) for non-normally distributed data. Two-tailed Student *t*-test or Mann–Whitney U test was used to compare continuous variables between groups depending on the normality of the data. The baseline characteristics were compared between included and excluded patients to evaluate the selection bias by our inclusion criteria.

Baseline characteristics had missing values in <5%, and these missing were considered to be due to missing completely at random. The incidences of missing in the baseline parameters were reported and were not replaced. There was no missing in our primary and secondary outcomes.

The following is the summary of our statistical analyses. Firstly, to evaluate the time-dependent change in the work index according to mortality, RVSWI values were compared between one-year survivors and nonsurvivors across the eight time-points. Moreover, as a post hoc analysis, we compared the time-dependent change in the two main components of RVSWI including right ventricle stroke volume index and MPAP. Mann–Whitney U test was used to compare the values at each time-point. Bonferroni correction was used to adjust multiple comparisons and *p* < 0.006 was considered as significant.

Secondly, Cox proportional hazard regression analysis was performed to evaluate whether time-weighted mean both ventricular stroke work indexes are independently associated with our primary outcome. We tested proportional hazard assumptions by visual inspection of log-minus-log survival plots for categorical variables and restricted cubic splines for continuous variables [18,19]. Before conducting multivariable analysis, multicollinearity among covariates was evaluated using the variance inflation factor. Variables with variance inflation factor >5 were excluded from the analysis. Cases with missing values of the covariates were excluded from Cox regression analysis for the complete case analysis. Other previously known predictors of poor outcomes including baseline CVP, baseline right ventricle end-diastolic volume, time-weighted mean PVR and mean Svo_2_ during anhepatic phase were included as covariates [2]. Time-weighted mean PVR and PVR at the end of surgery were included in the Cox regression analysis alternatively [5]. Stepwise variable selection process with backward Wald method using significance cutoff of 0.20 was performed. The calibration of Cox regression model was evaluated by Gronnesby and Borgan test [20]. The discrimination of Cox regression model was measured by Harrell’s C and Somers’ D [21]. To evaluate which component of RVSWI is significantly associated with mortality, we performed Cox regression analysis again after replacing the work index with time-weighted mean right ventricle stroke volume index and MPAP.

Thirdly, the following analyses were performed as planned sensitivity analyses. Our primary analysis of Cox regression was performed again with areas under the curve of both ventricular stroke work indexes and area under the curve of PVR to evaluate the time-dose response in terms of area under the curve. Kaplan–Meier survival analysis of our primary outcome was performed for the different time-weighted mean ventricular stroke work index groups to evaluate whether there is a significant difference in survival between the groups. Cubic spline function curve analysis was performed to evaluate the adjusted association between time-weighted mean both ventricular stroke work indexes, PVR and PVR at the end of surgery as continuous variables and one-year risk of death. Kaplan–Meier survival analysis and spline curve analysis were performed again with groups of areas under the curve of both work indexes and area under the curve of PVR. Detailed descriptions of these sensitivity analyses are reported in Supplemental Text S2.

Fourthly, post hoc analyses were performed to support the prognostic value of RVSWI. Detailed descriptions of these post hoc analyses are reported in Supplemental Text S2.

## 3. Results

Among the 1032 patients initially reviewed, 318 cases (30.8%) were excluded, because stroke work indexes could not be calculated, and 31 cases (3.0%) were excluded due to baseline hepatorenal syndrome. Among the remaining 683 cases that were finally included in our analysis, 65 (9.5%) died or experienced graft failure during one year after transplantation.

**Figure 1:**
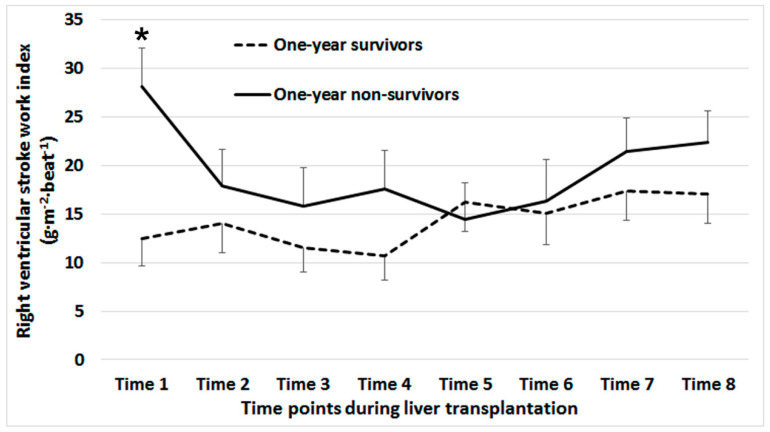
Time-dependent comparison of right ventricular stroke work index between one-year survivors and nonsurvivors. * Significant difference between groups (*p* < 0.001). Time 1: after anesthesia induction; Time 2: 1 h after anesthesia induction, Time 3: 10 min after the beginning of the anhepatic phase, 5 min before (Time 4) and after (Time 5) graft reperfusion; Time 6: 20 min after reperfusion; Time 7: 5 min after the completion of biliary reconstruction; Time 8: at the end of surgery.

Patient characteristics and perioperative variables are compared between intraoperative mean RVSWI groups in Table 1. The frequency of cases without missing was reported for all variables. In high RVSWI group, more patients have higher MELD scores and higher stages of Child–Turcotte–Pugh classification and received more amounts of colloid and transfusion. Our included and excluded patients had no significant difference in the baseline characteristics (Supplemental Table S1). Figure 1 shows time-dependent comparison of RVSWI between one-year survivors and nonsurvivors. There was a significant difference between groups at Time 1 (*p* < 0.001).

The results of Cox proportional hazard regression analysis for one-year all-cause mortality are shown in Table 2. The following variables were excluded from entering our multivariable model due to significant multicollinearity (variance inflation factor > 5) with other covariates: Child classification, Child–Turcotte–Pugh score, and fresh frozen plasma transfusion. All covariates met the proportional hazard assumptions. Time-weighted mean RVSWI was significantly associated with our primary outcome (hazard ratio 1.21, 95% confidence interval (CI) 1.12–1.36, *p* < 0.001). Our Cox regression model showed good calibration (Gronnesby and Borgan test: χ^2^ = 0.822, *p* = 0.391). Harrell’s C was 0.66, and Somers’ D was 0.51. When we replaced RVSWI with MPAP and right ventricle stroke volume index in the Cox regression model (*n* = 658, 96.3%), both right ventricle stroke volume index (hazard ratio 0.95, 95% CI 0.91–0.98, *p* < 0.001) and MPAP (hazard ratio 1.04, 95% CI 1.01–1.06, *p* = 0.015) were significantly associated with increased mortality (Supplemental Table S2).

**Figure 2:**
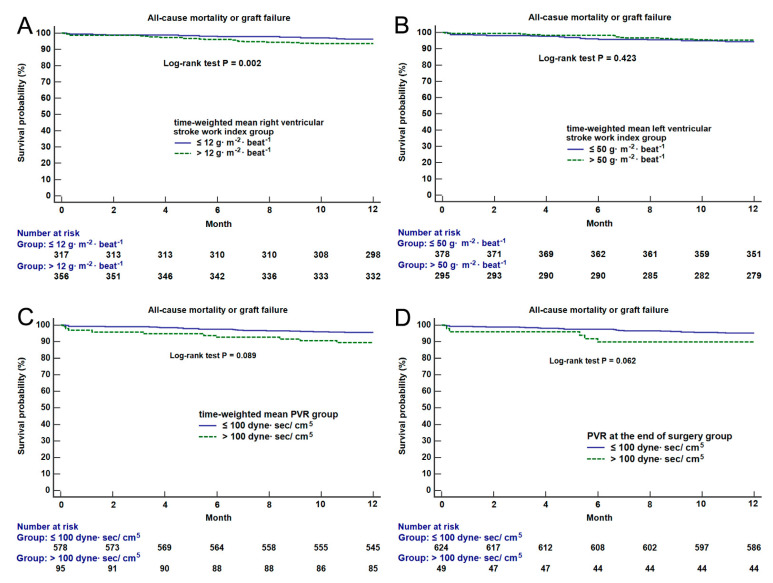
Kaplan–Meier survival curve analysis between intraoperative time-weighted mean right ventricular stroke work index groups (**A**), left ventricular stroke work index groups (**B**), time-weighted mean PVR groups (**C**) and PVR at the end of surgery groups (**D**). The results of log-rank test between the groups are shown on the figure. PVR = pulmonary vascular resistance.

Figure 2 shows the results of Kaplan–Meier survival curve analysis between groups. Mortality was significantly different between mean RVSWI groups (χ^2^ =12.7, *p* = 0.002). However, there was no significant difference in mortality between the groups of time-weighted left ventricular stroke work index, mean PVR and PVR at the end of surgery. The cause of death was compared between mean RVSWI groups (Supplemental Table S3). Mortalities related to cardiovascular complications were significantly more frequent in the high than low mean RVSWI group (*p* < 0.001).

**Figure 3:**
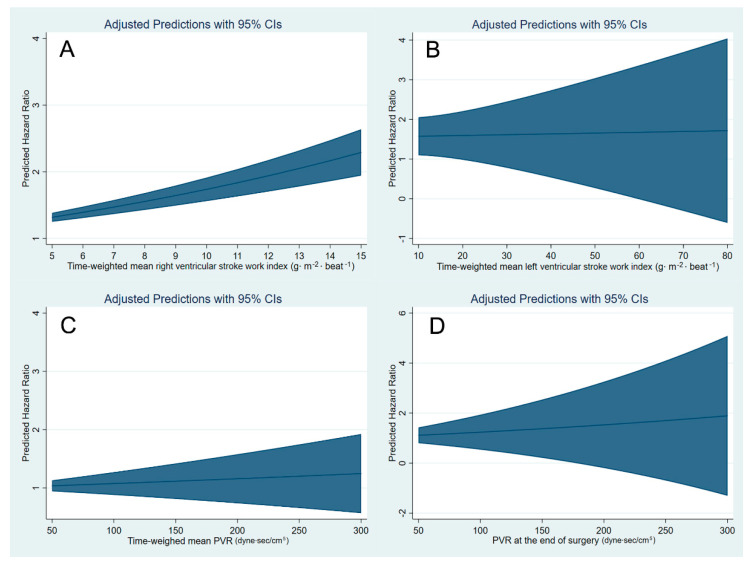
Cubic spline function curves of multivariable-adjusted relationship between intraoperative time-weighted mean right ventricular stroke work index (**A**), time-weighted mean left ventricular stroke work index (**B**) and time-weighted mean pulmonary vascular resistance (PVR) (**C**) and PVR at the end of surgery (**D**) as continuous variables and the risk of one-year all-cause mortality or graft failure.

The cubic spline function curves relating time-weighted mean both ventricular stroke work indexes, mean PVR and PVR at the end of surgery to the one-year risk of death or graft failure are shown in Figure 3. There was a gradual increase in predicted hazard ratio with a positive slope with time-weighted mean RVSWI, even in the normal reference range. Although hazard ratio also increases with PVR at the end of surgery, the slope was not as steep as that of mean RVSWI, and confidence interval was wide.

The results of logistic regression analysis for in-hospital mortality are shown in Supplemental Table S4. Time-weighted mean RVSWI was identified as an independent predictor of in-hospital mortality (Odds ratio 1.07, 95% CI 1.04–1.12, *p* < 0.001). Our regression model showed good calibration (χ^2^ =16.5, *p* = 0.821 by Hosmer–Lemeshow goodness of fit) and discrimination (Nagelkerke’s R^2^ = 0.432). When we replaced time-weighted mean values with area under the curve values, area under the curve of RVSWI was significantly associated with in-hospital mortality (Supplemental Table S5).

Postoperative secondary clinical outcomes were compared between the two time-weighted mean RVSWI groups. There were significant differences in the length of hospital and ICU stay, the incidence of acute kidney injury, and in-hospital mortality between groups (Table 3). Our sensitivity analysis of Cox regression model using area under the curve variables yielded consistent results. RVSWI was also significant when it was analyzed in terms of area under the curve (hazard ratio 1.24, 95% CI 1.15–1.37, *p* < 0.001) (Supplemental Table S6). Kaplan–Meier curve analyses were performed again with area under the curve groups of both ventricular stroke work indexes (Supplemental Figure S1). There was a significant difference between the area under the curve of RVSWI groups (χ^2^ = 22.3, *p* < 0.001). However, there was no significant difference between groups of area under the curve of left ventricular stroke work index (χ^2^ = 0.22, *p* = 0.832) or PVR (χ^2^ = 8.5, *p* = 0.140). The groups of baseline CVP (*p* = 0.080), baseline right ventricle end-diastolic volume (*p* = 0.123) or mean Svo_2_ during the anhepatic phase (*p* = 0.122) showed no significant difference (Supplemental Figure S2). The cubic spline curves for area under the curves of both ventricular stroke work indexes and PVR relating to one-year mortality were drawn (Supplemental Figure S3). There was a positive relationship only with area under the curve of RVSWI. Supplemental Figures S4 and S5 show the time-dependent comparison of MPAP and right ventricle stroke volume index between one-year survivors and nonsurvivors. There was a significant difference in MPAP at Time 1 (*p* < 0.001). There were significant differences in right ventricle stroke volume index at Time 5 (*p* = 0.002) and Time 6 (*p* = 0.005).

Propensity score matching yielded 263 pairs of high and low RVSWI group patients. All standardized differences of the covariates after matching were less than 0.1 (Supplemental Table S7), and their distributions before and after matching are shown in Supplemental Figure S6. The secondary clinical outcomes were compared between the matched high and low RVSWI groups. Significant differences in the length of hospital stay (*p* = 0.035), the incidence of acute kidney injury (*p* = 0.001) and in-hospital mortality (*p* = 0.005) were found (Table 3). There was a significant difference in survival between mean RVSWI groups in the matched cohort (χ^2^ = 15.6, *p* = 0.002) (Supplemental Figure S7).

The comparisons of clinical outcomes between the four groups of low–low, low–high, high–low and high–high groups are shown in Supplemental Table S8. There were significant differences between low–low and high–high groups in all outcome variables. Compared to the low–high group, the high–low group showed significantly better outcomes regarding length of hospital stay (*p* = 0.045) and one-year mortality (*p* = 0.002). Supplemental Figure S8 shows the results of Kaplan–Meier survival curve analysis between the four groups. There was a significant difference in survival between the four groups (Log-rank test, *p* < 0.001). The high–low group showed significantly better survival than the high–high group (*p* < 0.001) or the low–high group (*p* < 0.001).

## 4. Discussion

Our study evaluated the prognostic value of right heart-associated predictors including RVSWI to predict both in-hospital and one-year all-cause mortality or graft failure. Elevated intraoperative mean RVSWI was an independent predictor of mortality. Different RVSWI groups showed significant differences in survival up to one year after surgery. More patients with higher work index during surgery were associated with cardiovascular causes of death. Therefore, RVSWI could be a better prognostic tool to predict long-term survival after liver transplantation than PVR or other hemodynamic parameters. Both components of work index of stroke volume index and MPAP were significant predictors. Furthermore, given the results of our four-group analysis, intraoperative RVSWI could be a potentially modifiable hemodynamic goal to improve patient outcomes.

Preoperative hemodynamic status is associated with the clinical outcome of liver transplantation [22]. Intraoperative hemodynamic variables of right heart are associated with poor clinical outcomes [2,5]. Five-year survival of untreated portopulmonary hypertension was as low as 5.3% [23,24]. Right ventricle suffers hemodynamic challenges during liver transplantation. Frequent changes in preload by large fluid administration and transfusion or changes in afterload in advanced cirrhosis result in constant strain on the right ventricle. Among the right ventricle-associated hemodynamic parameters, intraoperative RVSWI is the amount of workload that the right ventricle actually suffers during surgery, while PVR is only a reflection of the static portion of afterload the right ventricle faces. Therefore, increased RVSWI may result in right heart dysfunction or failure after surgery, leading to graft congestion and potential impact on patient survival [25]. Additionally, high RVSWI may suggest intraoperative rapid and large administration of fluid reflecting excessive bleeding and surgical procedural difficulty. Conversely, low RVSWI may suggest right ventricular dysfunction, contributing to poor outcomes.

The causal relationship between RVSWI and mortality is unclear. However, patients with high work index were associated with frequent postoperative cardiovascular events in our data. Heart failure was considered to be a major cause of mortality after liver transplantation [26,27]. Notably, acute kidney injury occurred frequently in the high RVSWI group in our study. Since acute kidney injury could develop by decreased renal perfusion [28], RVSWI may contribute to mortality, at least in part, through increased risk of acute kidney injury.

RVSWI has two components of MPAP and right ventricle stroke volume index. Our post hoc Cox regression analysis showed that both components were significantly associated with mortality. However, our comparisons of time-dependent change in MPAP and right ventricle stroke volume index showed that high RVSWI in nonsurvivors was mainly due to increased MPAP. Since there were only ten patients with baseline portopulmonary hypertension in our patients, MPAP appears to increase newly intraoperatively. According to our calculation, 9.9% of our patients had average MPAP of >25 mmHg during surgery. These results suggest that intraoperative management of high MPAP might improve patient prognosis. However, time-dependent comparison of stroke volume index showed significant difference after reperfusion and neohepatic phase. Hemodynamic management according to stroke volume index could also be important.

RVSWI might be one of the modifiable hemodynamic goals of intraoperative hemodynamic management. According to our four-group analysis, intraoperative increase in work index was associated with increased mortality, while intraoperative decrease was associated with better outcomes. These results supported that RVSWI could be a modifiable risk factor. When high work index could be attributed to the high MPAP with high PVR, drugs for pulmonary hypertension including milrinone or nitrous oxide could be used [29,30,31]. However, the role of pulmonary vasodilators or inotropic support during liver transplantation is unclear. When MPAP was normal in patients with high RVSWI, high stroke volume index could be considered, which may be due to hyperdynamic circulatory status or volume overload.

Recently, intraoperative transesophageal echocardiography (TEE) is used during liver transplantation as a hemodynamic monitor to detect hypovolemia, myocardial ischemia, intracardiac air and hemodynamic events including pulmonary thromboembolism [1,32,33]. Real-time monitoring with TEE helps to evaluate the etiology of hemodynamic instability during each phase of liver transplantation [33,34]. Although we could not present intraoperative TEE data, we presented the preoperative baseline echocardiographic measurements (Supplemental Table S9). No difference in baseline findings between difference work index groups suggests the prognostic importance of intraoperative changes in the work index. The intraoperative TEE monitoring would help to evaluate the causes of altered RVSWI and right heart status. We developed an algorithm for the potential management of RVSWI according to other hemodynamic variables and TEE findings (Supplemental Figure S9), although specific treatment strategies should be validated in randomized trials. We recommend calculating RVSWI with simultaneous assessment of TEE findings according to our algorithm at the following time-points: after anesthesia induction as a baseline, during the anhepatic phase, after reperfusion and at the end of surgery.

Left ventricular stroke work index was not a significant predictor of patient and graft survival. In patients with advanced cirrhosis, a marked reduction in systemic vascular resistance and hyperdynamic hemodynamic status develop with tachycardia and increased cardiac output. Therefore, in cirrhotic patients, cardiac dysfunction is latent and only manifests under acute change in preload or afterload [35,36]. Reduced left ventricular contractility is usually masked by reduced arterial resistance. In this regard, left ventricular stroke work index may be less sensitive to detect the true cardiac workload.

A previous prospective study reported RVSWI in liver transplantation [6]. This study compared right heart-associated hemodynamic variables between those with and without portopulmonary hypertension. The intraoperative RVSWI after graft reperfusion was significantly greater in patients with baseline portopulmonary hypertension than those without. However, deterioration in right heart-associated hemodynamic parameters including RVSWI was not associated with perioperative mortality. This insignificance may be due to small sample size and not including the patients with severe portopulmonary hypertension.

The results of our study should be interpreted cautiously for several reasons. Firstly, this was a single-center retrospective cohort study. Hemodynamic management for the right heart dysfunction or pulmonary hypertension could not be standardized. Unknown or unmeasured confounders may have affected our analysis. External validity is limited due to the single-center design. Secondly, the causal relationship could not be established. However, we reported the cause of death and performed four-group analyses to assess whether intraoperative decreased RVSWI is associated with better outcomes. Thirdly, the patients without a pulmonary artery catheter or with hepatorenal syndrome were excluded from our analysis. Pulmonary artery catheter was not inserted in patients with hemodialysis catheter. However, there were no significant differences in other baseline characteristics between included and excluded patients. Fourthly, time-dependent comparison of RVSWI showed a significant difference only at the baseline value, questioning the importance of intraoperative monitoring. However, significant difference in stroke volume index after reperfusion and our four-group analysis supported the clinical relevance of intraoperative change.

## 5. Conclusions

In conclusion, intraoperative elevated RVSWI measured by time-weighted mean and area under the curve were associated with poor one-year all-cause mortality or graft survival after liver transplantation. RVSWI could be a hemodynamic management goal during liver transplantation. RVSWI could be an intraoperative hemodynamic goal during liver transplantation. However, the causal relationship between RVSWI and patient outcomes remains to be evaluated in further prospective trials.

## Figures and Tables

**Table 1:** Comparison of patient characteristics and perioperative parameters between low and high time-weighted mean right ventricular stroke work index (RVSWI) groups.

Characteristic	Low Right Ventricular Stroke Work Index Group (≤12 g·m^−2^·beat^−1^) *n* = 317	High Right Ventricular Stroke Work Index Group (>12 g·m^−2^·beat^−1^) *n* = 366	*p*-Value	% Cases with Complete Data
Demographic data				
Age, years	54 (50, 60)	52 (47, 58)	<0.001	100
Female, *n*	72 (22.7)	90 (24.6)	0.442	100
Body mass index, kg/m^2^	23.1 (21.4, 24.9)	23.3 (21.5, 25.4)	0.361	100
Etiology of liver disease				
Alcoholic liver cirrhosis, *n*	30 (9.5)	36 (9.8)	0.897	100
Hepatitis B viral hepatitis, *n*	156 (49.2)	110 (30.1)	<0.001	100
Hepatitis C viral hepatitis, *n*	24 (7.6)	27 (7.4)	0.923	100
Hepatocellular carcinoma, *n*	190 (59.9)	181 (49.5)	0.006	100
Cholestatic disease, *n*	7 (2.2)	8 (2.2)	>0.999	100
Nonalcoholic steatohepatitis, *n*	13 (4.1)	25 (6.8)	0.121	100
Baseline medical status				
Hypertension, *n*	39 (12.3)	32 (8.7)	0.128	100
Diabetes mellitus, *n*	46 (14.5)	36 (9.8)	0.061	100
Preoperative serum sodium, mEq/L	139 (135, 141)	138 (132, 140)	0.005	100
Preoperative hemoglobin, g/dL	11.9 (9.9, 13.5)	10.6 (9.2, 12.3)	<0.001	100
Preoperative serum albumin level, mg/dL	3.2 (2.7, 3.8)	2.9 (2.5, 3.4)	<0.001	100
Model for end-stage liver disease score	12 (8, 17)	15 (11, 22)	<0.001	98.4
Child–Turcotte–Pugh score	8 (5, 10)	8 (7, 11)	<0.001	100
Child classification, A/B/C, *n*	118 (37.2)/115 (36.3)/84 (26.5)	69 (18.9)/168 (45.9)/129 (35.2)	<0.001	97.7
Preoperative left ventricle ejection fraction, %	65 (60–69)	65 (61–68)	0.791	97.1
Preoperative portopulmonary hypertension, *n* *	-	10 (2.7)	-	96.6
Preoperative beta-blocker, *n*	18 (5.7)	16 (4.4)	0.483	98.4
Previous abdominal surgery, *n*	4 (1.3)	12 (3.3)	0.138	100
Donor/graft factors				
Age, years	30 (24, 39)	30 (23, 37)	0.127	100
Estimated graft-to-recipient weight ratio	1.20 (1.04, 1.43)	1.19 (1.04, 1.36)	0.181	98.8
Operation and anesthesia details				
Operation time, hour	425 (368, 490)	435 (388, 504)	0.062	100
Cold ischemic time, min	73 (64, 88)	72 (57, 88)	0.065	97.1
Warm ischemic time, min	31 (23, 39)	32 (24, 40)	0.355	97.1
Intraoperative mean PVR, dyne·sec/cm^5^	71 (56, 196)	80 (61, 244)	0.628	98.4
PVR at the end of surgery, dyne·sec/cm^5^	68 (42, 180)	78 (57, 225)	0.067	98.4
Intraoperative dose of epinephrine bolus, μg	0 (0, 15)	0 (0, 20)	0.937	99.6
Intraoperative mean blood glucose, mg/dL	163 (143, 180)	165 (149, 181)	0.111	99.9
Crystalloid administration, mL/kg	54 (36, 79)	53 (36, 75)	0.814	100
Colloid administration, mL/kg	0 (0, 9)	0 (0, 13)	0.002	100
Packed red blood cell transfusion, units	3 (0, 10)	5 (2, 10)	<0.001	100
Fresh frozen plasma transfusion, units	2 (0, 8)	6 (2, 10)	<0.001	100

The values are expressed as the median (25th, 75th percentiles) or number (%). PVR = pulmonary vascular resistance. * Portopulmonary hypertension was diagnosed when mean pulmonary artery pressure >25 mmHg.

**Table 2:** Multivariable Cox proportional hazard regression analysis to predict one-year all-cause mortality or graft failure after liver transplantation (*n* = 658).

Variable	Hazard Ratio (95% CI)	*p*-Value
Age, recipient	1.02 (1.01–1.05)	0.084
Female, recipient	0.51 (0.32–1.04)	0.069
Alcoholic liver cirrhosis	1.80 (0.80–3.72)	0.161
Hepatitis C viral hepatitis	1.58 (0.81–3.20)	0.240
Hepatocellular carcinoma	1.68 (1.15–2.45)	0.015
Preoperative hemoglobin, g/dL	0.81 (0.77–0.91)	0.001
Model for end-stage liver disease score	1.12 (1.03–1.15)	0.007
Warm ischemic time, min	0.97 (0.94–1.00)	0.105
Baseline right ventricle end-diastolic volume, mL	1.02 (1.00–1.03)	0.165
Mean Svo_2_ during anhepatic phase, %	0.96 (0.92–1.04)	0.325
Time-weighted mean of right ventricular stroke work index, g·m^−2^·beat^−1^	1.21 (1.12–1.36)	0.001
Time-weighted mean of left ventricular stroke work index, g·m^−2^·beat^−1^	1.05 (0.96–1.06)	0.214
Time-weighted mean of PVR	1.01 (0.99–1.02)	0.167
or PVR at the end of surgery, dyne·sec/cm^5^	1.02 (0.99–1.05)	0.085
Intraoperative mean blood glucose, mg/dL	1.01 (1.00–1.03)	0.156

CI = confidence interval, Svo_2_ = mixed venous oxygen saturation, PVR = pulmonary vascular resistance.

**Table 3:** Comparison of postoperative clinical outcomes between low and high time-weighted mean right ventricular stroke work index groups before and after propensity score matching.

	**Before Matching**		
**Variables**	**Low Right Ventricular Stroke Work Index Group** **(≤12 g·m^−2^·beat^−1^)**	**High Right Ventricular Stroke Work Index Group** **(>12 g·m^−2^·beat^−1^**)	***p*-Value**
Sample size	*n* = 317	*n* = 366	
Length of hospital stay, days	17 (14, 22)	20 (15, 26)	<0.001
Length of ICU stay, days	5 (4, 6)	5 (4, 7)	0.001
Acute kidney injury *, *n*	93 (29.3)	165 (45.1)	<0.001
In-hospital mortality, *n*	9 (2.8)	36 (9.8)	<0.001
One-year mortality or graft failure, *n*	17 (5.4)	48 (13.1)	<0001
	**After Matching**		
	**Low Right Ventricular Stroke Work Index Group** **(≤12 g·m^−2^·beat^−1^)**	**High Right Ventricular Stroke Work Index Group** **(>12 g·m^−2^·beat^−1^)**	***p*-Value**
Sample size	*n* = 263	*n* = 263	
Length of hospital stay, days	18 (15, 23)	20 (15, 26)	0.035
Length of ICU stay, days	5 (4, 6)	5 (4, 6)	0.096
Acute kidney injury *, *n*	82 (31.2)	118 (44.9)	0.001
In-hospital mortality, *n*	8 (3.0)	23 (8.7)	0.005
One-year mortality or graft failure, *n*	12 (4.6)	40 (15.2)	<0.001

Data are presented as median (interquartile range) or number (%). ICU = intensive care unit. * Determined during postoperative seven days and defined by the Kidney Disease Improving Global Outcomes serum creatinine criteria (≥1.5 times from baseline).

## Data Availability

The data presented in this study are available on request from the corresponding author.

## Supplementary Materials

The following are available online at https://www.mdpi.com/article/10.3390/jcm10174022/s1: Supplemental Text S1: Statistical analysis plan. Supplemental Text S2: Detailed statistical analysis of planned sensitivity analyses and post hoc analyses. Supplemental Figure S1: Kaplan–Meier survival curve analysis between groups of intraoperative area under the curve of right ventricular stroke work index (A), left ventricular stroke work index (B) and pulmonary vascular resistance (PVR) (C). The results of log-rank test between the groups are shown on the figure. Supplemental Figure S2: Kaplan–Meier survival curve analysis between groups of baseline central venous pressure (A), baseline right ventricular end-diastolic volume (B) and mean mixed venous oxygen saturation (Svo_2_) (C). The results of log-rank test between the groups are shown on the figure. Supplemental Figure S3: Cubic spline function curves of the multivariable-adjusted relationship between area under the curve of right ventricular stroke work index (A), area under the curve of left ventricular stroke work index (B) and area under the curve of pulmonary vascular resistance (PVR) (C) as continuous variables and the risk of one-year all-cause mortality or graft failure. Supplemental Figure S4: Time-dependent comparison of mean pulmonary artery pressure between one-year survivors and nonsurvivors. Supplemental Figure S5: Time-dependent comparison of right ventricle stroke volume index between one-year survivors and nonsurvivors. Supplemental Figure S6: Covariate balance plot showing the distribution of standardized differences between the two right ventricular stroke work index groups before and after propensity score matching. Supplemental Figure S7: Kaplan–Meier survival curve analysis between intraoperative time-weighted mean right ventricular stroke work index groups after propensity score matching. The result of log-rank test between the groups is shown on the figure. Supplemental Figure S8: Kaplan–Meier survival curve analysis between the four right ventricular stroke work index groups categorized by time-dependent change. The result of log-rank test between the groups is shown on the figure. Supplemental Figure S9: Suggested algorithm for hemodynamic management according to right ventricle stroke work index. Supplemental Table S1: Comparison of patient characteristics between the patients who were included and excluded from the analysis. Supplemental Table S2: Multivariable Cox proportional hazard regression analysis to predict one-year all-cause mortality or graft failure after liver transplantation after replacing right ventricular stroke work index with mean pulmonary artery pressure and right ventricle stroke volume index (*n* = 658). Supplemental Table S3: Cause of death during one-year follow-up after transplantation surgery. Supplemental Table S4: Multivariable logistic regression analysis to predict in-hospital all-cause mortality or graft failure after liver transplantation using time-weighted mean variables (*n* = 658). Supplemental Table S5: Multivariable logistic regression analysis to predict in-hospital all-cause mortality or graft failure after liver transplantation using area under curve of the variables (*n* = 658). Supplemental Table S6: Cox proportional hazard regression analysis to predict one-year all-cause mortality or graft failure after liver transplantation using area under curve of the variables (*n* = 658). Supplemental Table S7: Comparison of patient characteristics and perioperative parameters between low and high time-weighted mean right ventricular stroke work index groups before and after propensity score matching. Supplemental Table S8: Comparison of postoperative clinical outcomes between the four right ventricular stroke work index groups categorized by time-dependent change. Supplemental Table S9: Baseline echocardiographic findings between two intraoperative time-weighted mean right ventricular stroke work index groups.
