# Supplementary material for: The Prognostic Role of Right Ventricular Stroke Work Index during Liver Transplantation
Source: J Clin Med. 2021 Sep 6;10(17):4022. doi: 10.3390/jcm10174022 (PMC8432510; doi:10.3390/jcm10174022)
Supplement: Supplementary file 1 [file jcm-10-04022-s001.zip › jcm-1347088-supplementary.pdf]

Supplemental Materials

List	Title	Page
**Supplemental Text S1.**	Statistical analysis plan.	3
**Supplemental Text S2**.	Detailed statistical analysis of planned sensitivity analyses and post hoc analyses.	8
**Supplemental Figure S1**.	Kaplan-Meier survival curve analysis between groups of intraoperative area under the curve of right ventricular stroke work index (A), left ventricular stroke work index (B) and pulmonary vascular resistance (PVR) (C).	10
**Supplemental Figure S2**.	Kaplan-Meier survival curve analysis between groups of baseline central venous pressure (A), baseline right ventricular end-diastolic volume (B) and mean mixed venous oxygen saturation (Svo_2) (C).	11
**Supplemental Figure S3**.	Cubic spline function curves of the multivariable-adjusted relationship between area under the curve of right ventricular stroke work index (A), area under the curve of left ventricular stroke work index (B) and area under the curve of pulmonary vascular resistance (PVR) (C) as continuous variables and the risk of one year all-cause mortality or graft failure.	12
**Supplemental Figure S4.**	Time-dependent comparison of mean pulmonary artery pressure between one-year survivors and non-survivors.	13
**Supplemental Figure S5**.	Time-dependent comparison of right ventricle stroke volume index between one-year survivors and non-survivors.	14
**Supplemental Figure S6**.	Covariate balance plot showing the distribution of standardized differences between the two right ventricular stroke work index groups before and after propensity score matching.	15
**Supplemental Figure S7**.	Kaplan-Meier survival curve analysis between intraoperative time-weighted mean right ventricular stroke work index groups after propensity score matching.	16
**Supplemental Figure S8**.	Kaplan-Meier survival curve analysis between the four right ventricular stroke work index groups categorized by time-dependent change.	17
**Supplemental Figure S9**	Suggested algorithm for hemodynamic management according to right ventricle stroke work index.	18
**Supplemental Table S1.**	Comparison of patient characteristics between the patients who were included and excluded from the analysis.	19
**Supplemental Table S2.**	Multivariable Cox proportional hazard regression analysis to predict one-year all-cause mortality or graft failure after liver transplantation after replacing right ventricular stroke work index with mean pulmonary artery pressure and right ventricle stroke volume index (n=658).	20
**Supplemental Table S3.**	Cause of death during one-year follow-up after transplantation surgery.	21
**Supplemental Table S4.**	Multivariable logistic regression analysis to predict in-hospital all-cause mortality or graft failure after liver transplantation using time-weighted mean variables (n=658).	22
**Supplemental Table S5.**	Multivariable logistic regression analysis to predict in-hospital all-cause mortality or graft failure after liver transplantation using area under curve of the variables (n=658).	23
**Supplemental Table S6.**	Cox proportional hazard regression analysis to predict one-year all-cause mortality or graft failure after liver transplantation using area under curve of the variables (n=658).	24
**Supplemental Table S7.**	Comparison of patient characteristics and perioperative parameters between low and high time-weighted mean right ventricular stroke work index groups before and after propensity score matching.	25
**Supplemental Table S8.**	Comparison of postoperative clinical outcomes between the four right ventricular stroke work index groups categorized by time-dependent change.	26
**Supplemental Table S9**	Baseline echocardiographic findings between two intraoperative time-weighted mean right ventricular stroke work index groups.	27

Summary of our statistical analysis

Outcome	Independent varible	Analysis	Statistics	Results
1-year all-cause mortality and graft failure	Time-weighted mean RVSWI	Primary analysis	Multivariable Cox proportional hazard analysis	Table 2
			Kaplan-Meier survival curve analysis	Figure 2
			Cubic spline function curve analysis	Figure 3
	Area under the curve of RVSWI	Sensitivity analysis	Multivariable Cox proportional hazard analysis	Supplemental Table S6
			Kaplan-Meier survival curve analysis	Supplemental Figure S1
			Cubic spline function curve analysis	Supplemental Figure S3
In-hospital all-cause mortality and graft failure	Time-weighted mean RVSWI	Secondary analysis	Multivariable logistic regression analysis	Supplemental Table S4
	Area under the curve of RVSWI		Multivariable logistic regression analysis	Supplemental Table S5
Baseline analysis	Time-weighted mean RVSWI		Propensity score matching analysis – comparison of baseline characteristics	Supplemental Table S7
Baseline analysis			Propensity score matching analysis - covariate balance plot	Supplemental Figure S6
Secondary clinical outcomes			Propensity score matching analysis – comparison of outcomes	Supplemental Table S8
1-year all-cause mortality and graft failure			Propensity score matching analysis - Kaplan-Meier survival curve analysis	Supplemental Figure S7
1-year all-cause mortality and graft failure and secondary outcomes	Four groups by time-dependent change		Comparison of postoperative clinical outcomes	Supplemental Table S8
1-year all-cause mortality and graft failure			Kaplan-Meier survival curve analysis	Supplemental Figure S8

Supplemental Text S1. Prognostic value of right and left ventricular stroke work index in liver transplantation - Statistical analysis plan
Principle investigator: Won Ho Kim
Co-Investigators: Seong-Mi Yang, Ho-Jin Lee
Affiliation: Department of Anesthesiology and Pain Medicine, Seoul National University Hospital, Seoul National University College of Medicine
Version number: 1.0.
Reviewed and approved by: Seoul National University Institutional Review Board
Institutional Review Board registration number: 1904-118-1028
Date of Approval: 29, April 2019
This document reports the statistical analysis plan for a retrospective observational cohort study entitled with "Prognostic value of right and left ventricular stroke work index in liver transplantation". This document was originally submitted for review by Seoul National University Institutional Review Board.

I. Data collection
This study is an observational study for a retrospective cohort of the patients undergoing elective living-donor liver transplantation. We will review and screen the electronic medical records of 1032 consecutive adult patients who underwent elective living donor liver transplantation between 2004 and 2015. According to the exclusion criteria, remaining patients will be included in our data analysis.
Demographic or perioperative variables known to be related to postoperative graft survival or patient mortality will be collected.
(1) Recipient demographics
Age, sex, body-mass index
(2) Etiology of liver disease
Hepatitis B viral hepatitis, Hepatitis C viral hepatitis, hepatocellular carcinoma, cholestatic disease, non-alcoholic steatohepatitis, alcoholic liver cirrhosis
(3) Baseline medical condition
Past medical history: hypertension, diabetes mellitus, chronic kidney disease
Previous abdominal surgery
Model for End-stage Liver Disease score, Child-Turcotte-Pugh score, Child classification (A/B/C)
Presence of hepatorenal syndrome, portopulmonary hypertension
(4) Baseline laboratory findings
Preoperative hemoglobin, albumin levels, serum sodium concentration
Preoperative echocardiography: left ventricle ejection fraction (%)
(5) Donor-related variables
Age, sex, body-mass index, estimated graft recipient body-weight ratio
(6) Operation-related variables
Cold ischemic time, warm ischemic time
(7) Anesthesia-related variables
Insertion of pulmonary artery catheter, main anesthetic agent, intraoperative dose of epinephrine, intraoperative mean blood glucose, packed Red Blood Cell transfusion, Fresh Frozen Plasma transfusion, amount of crystalloid and colloid administration
(8) Hemodynamic parameters
Right heart-associated variables: central venous pressure (mmHg), right ventricle end-diastolic volume (ml), Right ventricle stroke index ($ml \cdot beat^{-1} \cdot m^{-2}$), mean pulmonary artery pressure, pulmonary vascular resistance (PVR, dyne·sec/cm^5), Mixed venous oxygen saturation (Svo_2, %)
Left ventricle associated variables: mean arterial pressure (mmHg), cardiac index ($ml \cdot beat^{-1} \cdot m^{-2}$), systemic vascular resistance (SVR, dyne·sec/cm^5)
These variables will be collected at eight times during surgery: (Time 1) after anesthesia induction, (Time 2) 1 hour after anesthesia induction, (Time 3) 10 min after the beginning of the anhepatic phase, (Time 4) 5 min before and (Time 5) after graft reperfusion, (Time 6) 20 min after reperfusion, (Time 7) 5 min after the completion of biliary reconstruction, and (Time 8) at the end of surgery.
(9) Calculation of right and left ventricular stroke work index
Right ventricle stroke work index and left ventricle stroke work index will be calculated by the following equation at eight time-points during liver transplantation.
Right ventricular stroke work index ($g \cdot m^{-2} \cdot beat^{-1}$) [normal reference value: 5-10 $g \cdot m^{-2} \cdot beat^{-1}$]
= Right ventricule stroke volume index ($mL \cdot beat^{-1} \cdot m^{-2}$) × [mean pulmonary arterial pressure (mmHg) −

central venous pressure (mmHg)] × 0.0136

Left ventricular stroke work index ($g \cdot m^{-2} \cdot beat^{-1}$) [normal reference value: 45-60 $g \cdot m^{-2} \cdot beat^{-1}$]

$= \text{Left ventricle stroke volume index } (mL \cdot beat^{-1} \cdot m^{-2}) \times [\text{mean arterial pressure (mmHg)} - \text{pulmonary artery occlusion pressure (mmHg)}] \times 0.0136$

Eight times during surgery: (Time 1) after anesthesia induction, (Time 2) 1 hour after anesthesia induction, (Time 3) 10 min after the beginning of the anhepatic phase, (Time 4) 5 min before and (Time 5) after graft reperfusion, (Time 6) 20 min after reperfusion, (Time 7) 5 min after the completion of biliary reconstruction, and (Time 8) at the end of surgery.

These eight time-points were determined to collect hemodynamic variables systemically for our institutional database of liver transplantation. The time points were selected when can be used as a baseline or when surgical phase changes during liver transplantation or when the hemodynamic instabilities are expected according to our experiences.

(10) Right and left ventricular stroke work index-associated secondary variables

With right ventricular stroke work index and left ventricular stroke work index measured at eight times during surgery, the following secondary variables will be calculated [1]. Two investigators (WHK and SY) will independently calculate the right ventricular stroke work index and left ventricular stroke work index-related variables and will compare the calculated values. Any discrepancies will be resolved by repeated calculation.

- **Time-weighted mean right ventricular stroke work index, left ventricular stroke work index, PVR**

Time-weighted mean right ventricular stroke work index and left ventricular stroke work index will be calculated according to the following equation [2].

$$\text{Time} - \text{weighted mean} = \frac{[(X_1 + X_2)(T_2 - T_1) + (X_2 + X_3)(T_3 - T_2) + \cdots + (X_{n-1} + X_n)(T_n - T_{n-1})]}{2 \times (T_n - T_1)}$$

(T_n, time of measurement of X_n; X_n = right ventricular stroke work index or left ventricular stroke work index at T_n).

- **Area under curve of right ventricular stroke work index, left ventricular stroke work index, PVR**

Area under curve will be calculated by the trapezium rule according to the following reference [3].

Matthews JN, Altman DG, Campbell MJ, Royston P: Analysis of serial measurements in medical research. BMJ 1990; 300:230-5

II. Definitions of the study outcome variables

The primary outcome is **all-cause mortality or graft failure requiring retransplantation during one years after liver transplantation.**

Mortality data source:

The date and cause of death of all patients including those lost to follow-up will be collected from the Korean national statistics service (http://kostat.go.kr/portal/eng).

Graft failure data source:

The date of graft failure will be determined according to the review of medical chart.

Secondary postoperative clinical outcomes will be postoperative hospital length of stay, and intensive care unit length of stay, in-hospital mortality, postoperative acute kidney injury defined by Kidney disease; improving global outcomes criteria (KDIGO) using serum creatinine during postoperative seven days [4].

KDIGO serum creatinine criteria	
**Stage 1**	≥ 0.3 mg/dL increase within 48hrs<br>or **≥ 1.5 fold increase** from baseline within 7 days
**Stage 2**	**≥ 2 fold increase** from baseline within 7 days
**Stage 3**	**≥ 3 fold increase** from baseline within 7 days<br>or increase to 4.0 mg/dL with an acute increase of 0.5 mg/dL<br>or Any requirements for renal replacement therapy

III. Statistical analysis

1. Statistical software used for the statistical analysis

· STATA/MP version 15.1 (StataCort. College Station, TX, USA) – for any regression analysis and cubic spline function curve analysis

· Medcalc Statistical Software version 18.6 (MedCalc Software bvba, Ostend, Belgium) – for depicting survival curve analysis
· SPSS software version 25.0 (IBM Corp., Armonk, NY, USA) – for the remaining statistical analyses.

2. Handling missing values and outliers
The incidence of missing values for the baseline characteristics, outcome variables and lost to follow-up will be reported.
The multivariable Cox regression analysis will be conducted with the complete case analysis.
Possible outliers will be examined by one of our investigator (WHK) and will be excluded from our analysis if outliers are regarded as definite.

3. Power calculation
The sample size will be based on the available data from all patients who underwent liver transplantation at our institution from 2004 and 2015 and met our inclusion criteria.
No statistical power calculation will be performed prior to the study.

4. Reporting the values
Categorical variables will be reported as number (percentage). The normality of the data will be determined by the Shapiro-Wilk test or visual inspection of quantile-quantile plot. Continuous variables will be presented as mean ± SD for normally distributed data or median [interquartile range, 25th and 75th percentile] for non-normally distributed data.

5. Comparison of time-dependent distribution of right ventricular stroke work index or left ventricular stroke work index values
Comparison of time-dependent distribution of right ventricular stroke work index or left ventricular stroke work index between one-year survivors and non-survivors at all eight time points. The investigators will draw figures showing time-dependent changes in right ventricular stroke work index or left ventricular stroke work index if these parameters are revealed to be significant predictors of mortality or graft failure.

6. Categorizing and grouping time-weighted mean right ventricular stroke work index or left ventricular stroke work index values
We will divide the patients into two groups according to the time-weighted mean right ventricular stroke work index or left ventricular stroke work index values and compare the baseline characteristics between them.
Either right ventricular stroke work index or left ventricular stroke work index will be chosen depending on the statistical significance of being significant predictor of mortality or graft failure. The cutoff used for dividing right ventricular stroke work index or left ventricular stroke work index groups will be their median values of our study population.

7. Comparison of variables between baseline characteristics between groups
Two-tailed Student t-test or Mann-Whitney U test will be used to compare continuous variables between groups depending on the normality of the data. Chi-square test or Fisher's exact test will be used to compare categorized variables between groups depending on their expected counts.

8. Cox proportional hazard regression analysis: Primary analysis for primary endpoint
Cox proportional hazard regression analysis will be performed to evaluate whether time-weighted mean right ventricular stroke work index and left ventricular stroke work index are independently associated with one-year mortality or graft failure.
Cox regression will be conducted by complete case analysis: the complete cases without missing values will be included in the Cox regression analysis.
The proportional hazard assumption will be checked by visual inspection of log-minus-log survival plots for categorical variables and restricted cubic splines for continuous variables [5,6].
Before conducting multivariable analysis, multicollinearity among covariates will be assessed using the variance inflation factor. A covariate with a variance inflation factor greater than 5 will be excluded from the analysis.
Other previously-known predictors of poor outcomes including time-weighted mean PVR, baseline CVP, baseline right ventricle end-diastolic volume and mean Svo_2 during anhepatic phase will be included as

covariates [7]. Mean PVR and PVR at the end of surgery will be included in the analysis alternatively [8].
All baseline and intraoperative variables will be adjusted in the multivariable Cox regression analysis using the enter method.
As a sensitivity analysis, variable selection process of backward Wald method will be used with significant criterion of 0.20.
The performance of Cox regression model will be measured by Harrell's c and Somers' D [9] (by STATA) [10].
The calibration of Cox regression model will be evaluated by Groennesby and Borgan test (by STATA, stcox-gof function).
A priori effect sizes (hazard ratio) of the exposure variables for the Cox regression analysis for one-year all-cause mortality or graft failure of our study were determined as follows.
Time-weighted mean right ventricular stroke work index: 1.10
Time-weighted mean left ventricular stroke work index: 1.10
Time-weighted mean PVR: 1.05
PVR at the end of surgery: 1.05

9. Kaplan-Meier survival analysis
Kaplan-Meier survival curve analysis of one-year mortality or graft failure will be performed for mean right ventricular stroke work index and mean left ventricular stroke work index groups.
Patients will be divided into two groups of mean right ventricular stroke work index and left ventricular stroke work index using cutoffs as the medians of observed mean right ventricular stroke work index or left ventricular stroke work index.
The log-rank test will be used to determine statistical significance between groups.
For the survival analysis of other hemodynamic variables, the same analysis for baseline central venous pressure, baseline right ventricle end-diastolic volume and mean mixed venous oxygen saturation (Svo_2) during anhepatic phase will be performed [7].

10. Cubic spline function curve analysis
Cubic spline function curves will be drawn by STATA to evaluate the adjusted relationship between time-weighted mean right ventricular stroke work index, time-weighted mean left ventricular stroke work index, time-weighted mean PVR and PVR at the end of surgery as continuous variables and one-year risk of death [11-13].

11. Comparison of the cause of death between the time-weighted mean right ventricular stroke work index or left ventricular stroke work index groups.
Liver disease-related (hepatic failure, recurrent hepatocellular carcinoma) or cardiovascular causes of death (e.g. heart failure, pulmonary thromboembolism, cerebrovascular accident) will be investigated to evaluate the potential causal relationship between the hemodynamic variables of right ventricular stroke work index and left ventricular stroke work index and mortality. The cause of death will be compared between different right ventricular stroke work index or left ventricular stroke work index groups if right ventricular stroke work index or left ventricular stroke work index were identified to be a significant predictor of mortality.

12. Sensitivity Analysis plan
- Cox regression analysis will be performed again with area under curve of right ventricular stroke work index, area under curve of left ventricular stroke work index and area under curve of PVR.
- Kaplan-Meier analysis will be performed again for groups of area under curve of right ventricular stroke work index, area under curve of left ventricular stroke work index and area under curve of PVR. For area under curve variables, cutoff points will be determined as the median for area under curve of right ventricular stroke work index, area under curve of left ventricular stroke work index, and area under curve of PVR.
- Cubic spline function curve analysis will be performed for area under curve of right ventricular stroke work index, area under curve of left ventricular stroke work index and area under curve of PVR.

13. Post-hoc analysis: Comparison of patient characteristics between included and excluded patients
The demographics, etiology of liver disease, baseline medical status, and donor/ graft factors will be compared between the patients who will be included and excluded from the analysis. This analysis is to evaluate the potential selection bias caused by our inclusion and exclusion criteria.

References

[1] Yilmaz HO, Babazade R, Leung S, Zimmerman NM, Makarova N, Saasouh W, Stocchi L, Gorgun E, Sessler DI, Turan A: Postoperative Hypotension and Surgical Site Infections After Colorectal Surgery: A Retrospective Cohort Study. Anesth Analg 2018; 127:1129-36.

[2] Salmasi V, Maheshwari K, Yang D, Mascha EJ, Singh A, Sessler DI, Kurz A: Relationship between Intraoperative Hypotension, Defined by Either Reduction from Baseline or Absolute Thresholds, and Acute Kidney and Myocardial Injury after Noncardiac Surgery: A Retrospective Cohort Analysis. Anesthesiology 2017; 126:47-65.

[3] Matthews JN, Altman DG, Campbell MJ, Royston P: Analysis of serial measurements in medical research. Bmj 1990; 300:230-5.

[4] Thomas ME, Blaine C, Dawnay A, Devonald MA, Ftouh S, Laing C, Latchem S, Lewington A, Milford DV, Ostermann M: The definition of acute kidney injury and its use in practice. Kidney Int 2015; 87:62-73.

[5] Govindarajulu US, Spiegelman D, Thurston SW, Ganguli B, Eisen EA: Comparing smoothing techniques in Cox models for exposure-response relationships. Stat Med 2007; 26:3735-52.

[6] Durrleman S, Simon R: Flexible regression models with cubic splines. Stat Med 1989; 8:551-61.

[7] Kim WH, Oh HW, Yang SM, Yu JH, Lee HC, Jung CW, Suh KS, Lee KH: Intraoperative Hemodynamic Parameters and Acute Kidney Injury After Living Donor Liver Transplantation. Transplantation 2019; 103:1877-86.

[8] Park J, Lee SH, Kim J, Park SJ, Park MS, Choi GS, Lee SK, Kim GS: Predictive Value of Intraoperative Pulmonary Vascular Resistance in Liver Transplantation. Liver Transpl 2018; 24:1680-9.

[9] Harrell FE, Jr., Lee KL, Mark DB: Multivariable prognostic models: issues in developing models, evaluating assumptions and adequacy, and measuring and reducing errors. Stat Med 1996; 15:361-87.

[10] Newson R: Confidence intervals for rank statistics: Somers' D and extensions. Stata Journal 2006; 6:309-34.

[11] Helwig NE, Shorter KA, Ma P, Hsiao-Wecksler ET: Smoothing spline analysis of variance models: A new tool for the analysis of cyclic biomechanical data. J Biomech 2016; 49:3216-22.

[12] Du P, Jiang Y, Wang Y: Smoothing spline ANOVA frailty model for recurrent event data. Biometrics 2011; 67:1330-9.

[13] Kirby S, Colman P, Morris M: Adaptive modelling of dose-response relationships using smoothing splines. Pharm Stat 2009; 8:346-55.

Supplemental Text S2. Detailed statistical analysis of planned sensitivity analyses and post hoc analyses.

The followings are detailed description of our planned sensitivity analyses.

Firstly, our primary analysis of Cox proportional hazard regression analysis was performed again with area under curve of right ventricular stroke work index, area under curve of left ventricular stroke work index and area under curve of pulmonary vascular resistance (PVR) to evaluate the time-dose response in terms of area under curve.

Secondly, Kaplan-Meier survival analysis of one-year mortality or graft failure was performed for the mean right ventricular stroke work index and mean left ventricular stroke work index groups. Our survival data were right-censored for the survival analysis. Patients were divided into two groups of mean right ventricular stroke work index and left ventricular stroke work index using cutoffs of 12 and 50 $g \cdot m^{-2} \cdot beat^{-1}$, respectively. The cutoffs were determined as the medians of observed mean right ventricular stroke work index or left ventricular stroke work index. We used medians as cutoffs because there was no previously-reported optimal reference range during liver transplantation and these cutoffs were close to the upper normal reference range of the general population [1]. The log-rank test was used to determine statistical significance between groups. As a sensitivity analysis, Kaplan-Meier analysis was performed again for groups of area under curve of right ventricular stroke work index, area under curve of left ventricular stroke work index and area under curve of PVR. For area under curve variables, cutoff points were determined as their medians (4 $g \cdot m^{-2} \cdot beat^{-1} \times 24$ hour for right ventricular stroke work index, 14 $g \cdot m^{-2} \cdot beat^{-1} \times 24$ hour for left ventricular stroke work index, 30 $dyne \cdot sec/m^5 \times 24$hour for PVR). The same analysis for baseline central venous pressure, baseline right ventricle end-diastolic volume and mean Svo_2 during anhepatic phase were performed. The causes of death during one-year follow-up were compared between groups of time-weighted mean right ventricular stroke work index to evaluate the possible causal relationship between high right ventricular stroke work index and mortality.

Thirdly, we performed the cubic spline function curve analysis to evaluate the adjusted association between time-weighted mean right ventricular stroke work index, left ventricular stroke work index, PVR, and PVR at the end of surgery as continuous variables and one-year risk of death. Also, spline curve analysis was performed for area under curves of right ventricular stroke work index, left ventricular stroke work index, and PVR.

The followings are our post hoc analyses to support the prognostic value of ventricular stroke work index.

Firstly, we performed a multivariable binary logistic regression analysis for in-hospital mortality to assess whether right ventricular stroke work index is also an independent predictor of short-term outcomes. We included the same covariates used in the Cox regression analysis and used stepwise variable selection process with backward Wald method using significance cutoff of 0.20. The calibration and discrimination of the resulting regression model were evaluated by Hosmer-Lemeshow goodness of fit and Nagelkerke's R^2, respectively.

Secondly, propensity score analysis was performed to reduce the potential confounding effect of baseline differences between the two mean right ventricular stroke work index groups. The propensity score was defined as the probability of being included in the high or low right ventricular stroke work index groups by logistic regression analysis. The contributors to the propensity score included all demographic data, etiology of liver disease, baseline medical status, and donor/ graft factors listed in Table 1. All covariates were matched at a ratio of 1:1 using the nearest neighborhood method with a caliper width of 0.1. To determine balance between the two groups, we compared absolute standardized differences before and after matching. An absolute standardized difference of less than 0.1 was considered that the two groups were balanced for the covariates. In the matched cohort, we compared our secondary study outcomes. Kaplan-Meier survival analysis of one-year mortality or graft failure were performed again for the mean right ventricular stroke work index groups in the matched cohort.

Thirdly, we evaluated whether the time-dependent changes in right ventricular stroke work index between anesthesia induction and the end of surgery could affect our study outcomes. Patients were classified as four groups of low-low (right ventricular stroke work index after anesthesia induction ≤12 $g \cdot m^{-2} \cdot beat^{-1}$; right ventricular stroke work index at the end of surgery ≤12 $g \cdot m^{-2} \cdot beat^{-1}$), low-high, high-low, and high-high groups. Then, the mortality rate and secondary clinical outcomes were compared between groups. Kaplan-Meier survival curve analysis was performed and log-rank test was used to compare overall survival between groups.

Fourthly, to evaluate the relative importance of the different predictor variables including right and left ventricular stroke work indexes, we calculated standardized regression coefficient after standardizing

the continuous covariates of the multivariable Cox regression analysis for our primary outcome. Continuous variables were standardized by dividing the difference between the individual value and the mean by the standard deviation.

Finally, to evaluate whether liver transplantation-related increased right ventricular stroke work index or pre-surgical increased right ventricular stroke work index regardless of surgery type is responsible for one-year higher mortality, we performed Cox regression analysis and Kaplan-Meier survival analysis in patients undergoing cardiac surgery under cardiopulmonary bypass. Detailed methods were reported in Supplemental Text S2.

Reference

[1] Reich D, Mittnacht A, Manecke JR G, Kaplan J: Monitoring of the heart and vascular system, Kaplan's cardiac anesthesia: the echo era, 6th edition. Edited by Kaplan J. St. Louis, Missouri, Elsevier, 2011, pp 433

Supplemental Figure S1. Kaplan-Meier survival curve analysis between groups of intraoperative area under the curve of right ventricular stroke work index (A), left ventricular stroke work index (B) and pulmonary vascular resistance (PVR) (C). The results of log-rank test between the groups are shown on the figure.

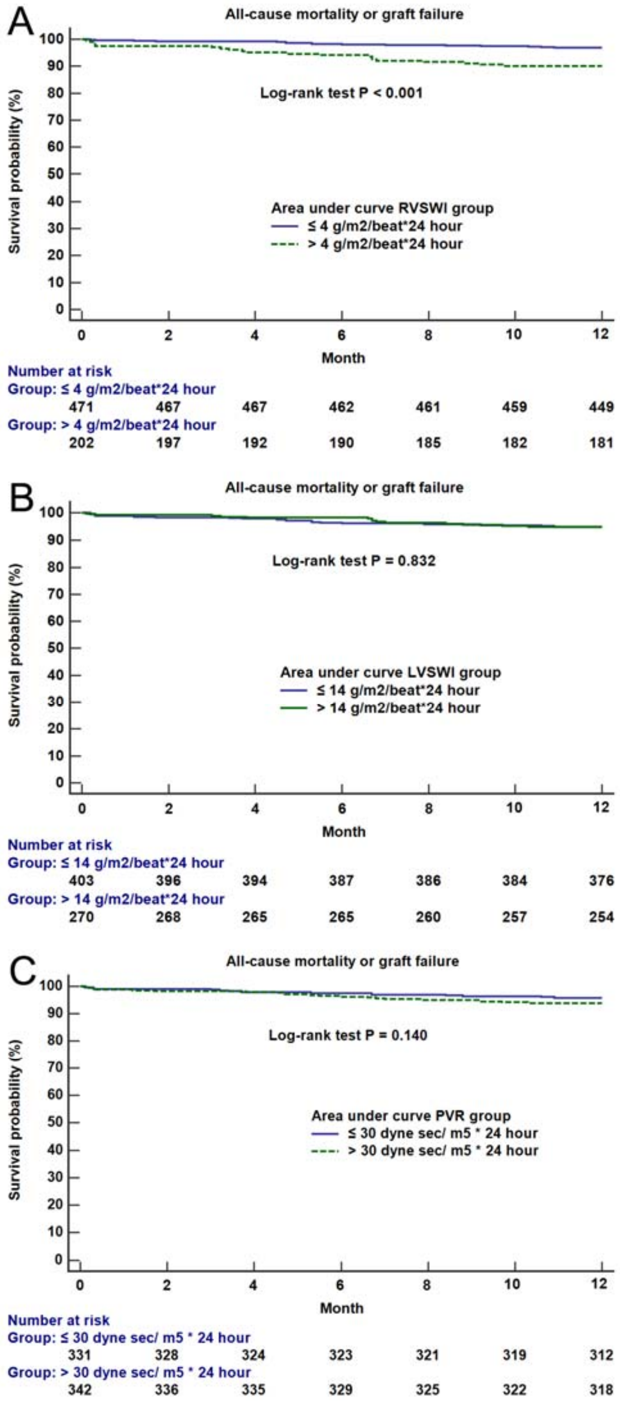

Supplemental Figure S2. Kaplan-Meier survival curve analysis between groups of baseline central venous pressure (A), baseline right ventricular end-diastolic volume (B) and mean mixed venous oxygen saturation (Svo2) (C). The results of log-rank test between the groups are shown on the figure.

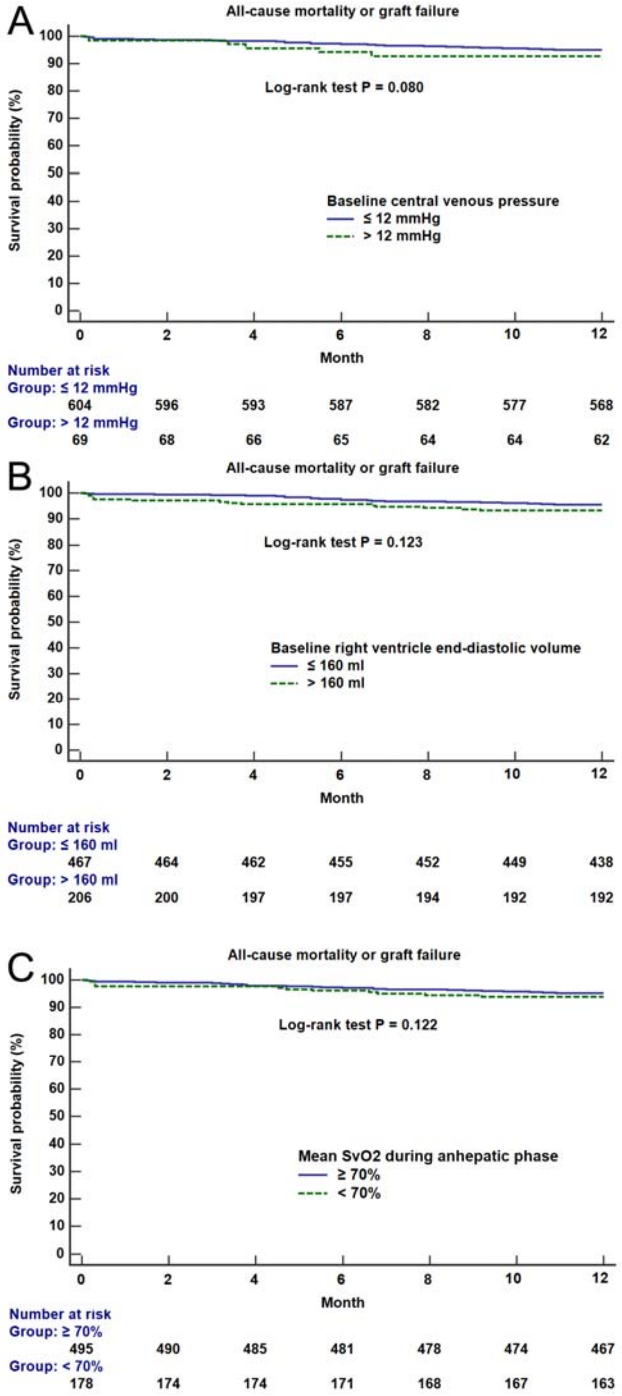

Supplemental Figure S3. Cubic spline function curves of the multivariable-adjusted relationship between area under the curve of right ventricular stroke work index (RVSWI) (A), area under the curve of left ventricular stroke work index (LVSWI) (B) and area under the curve of pulmonary vascular resistance (PVR) (C) as continuous variables and the risk of one year all-cause mortality or graft failure.

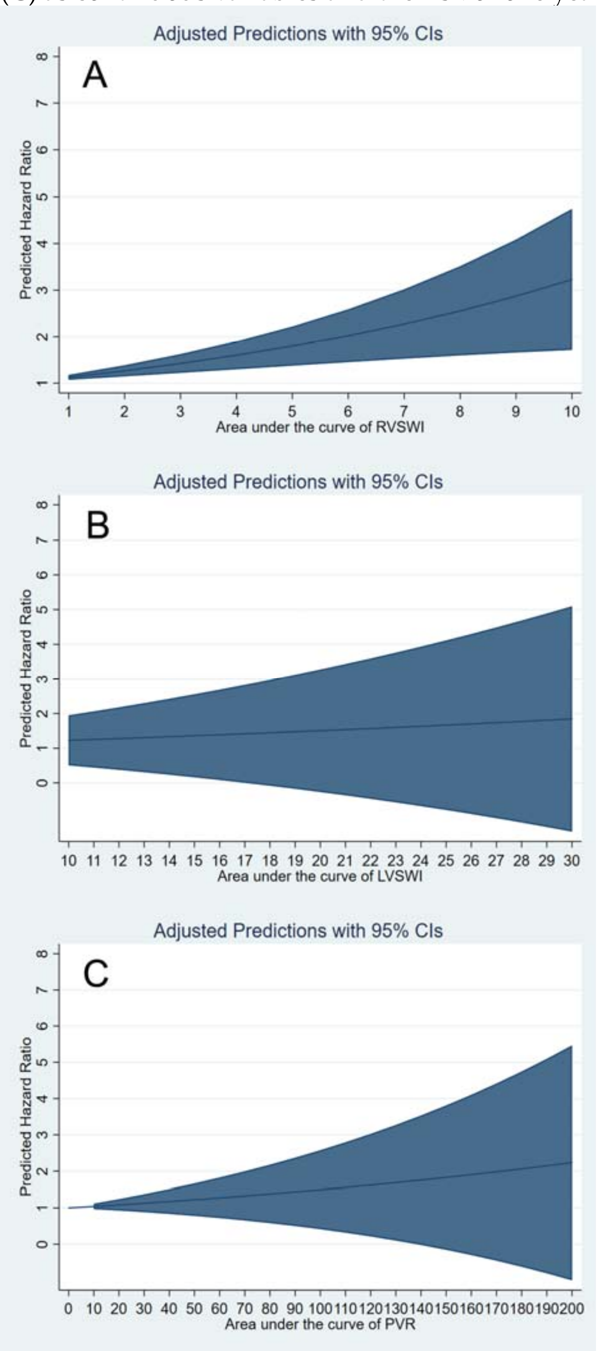

Supplemental Figure S4. Time-dependent comparison of mean pulmonary artery pressure between one-year survivors and non-survivors. *Significant difference between groups. Time 1: after anesthesia induction, Time 2: 1 hour after anesthesia induction, Time 3: 10 min after the beginning of the anhepatic phase, 5 min before (Time 4) and after (Time 5) graft reperfusion, Time 6: 20 min after reperfusion, Time 7: 5 min after the completion of biliary reconstruction, Time 8: at the end of surgery.

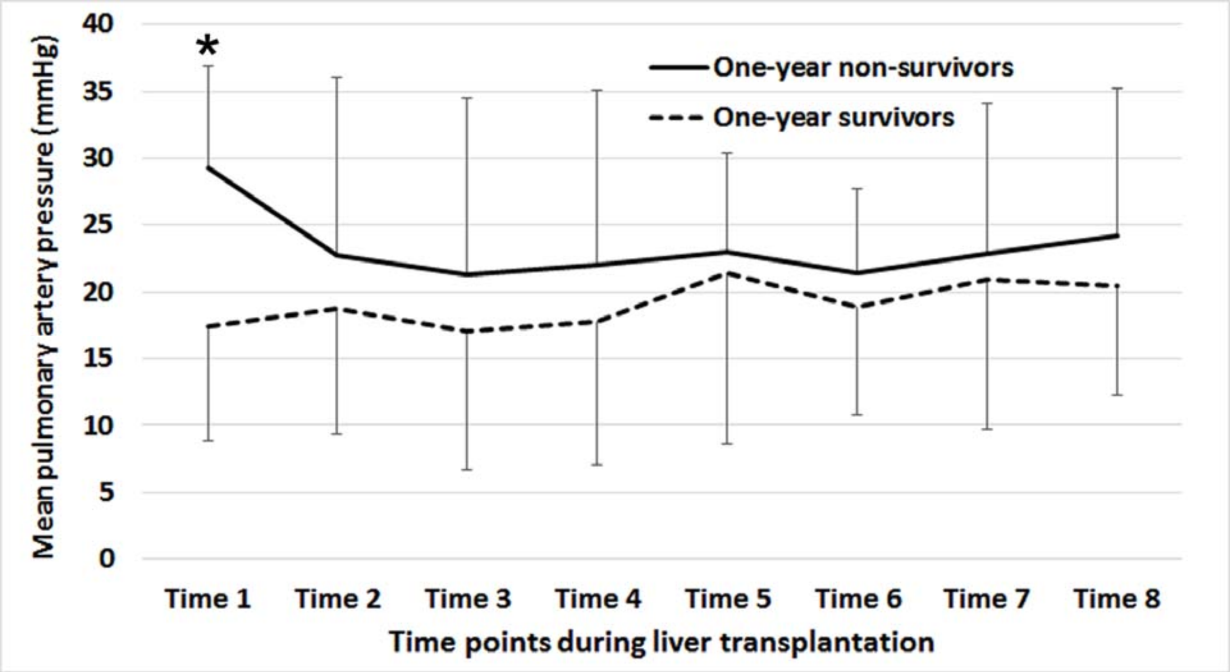

Supplemental Figure S5. Time-dependent comparison of right ventricle stroke volume index between one-year survivors and non-survivors. *Significant difference between groups. Time 1: after anesthesia induction, Time 2: 1 hour after anesthesia induction, Time 3: 10 min after the beginning of the anhepatic phase, 5 min before (Time 4) and after (Time 5) graft reperfusion, Time 6: 20 min after reperfusion, Time 7: 5 min after the completion of biliary reconstruction, Time 8: at the end of surgery.

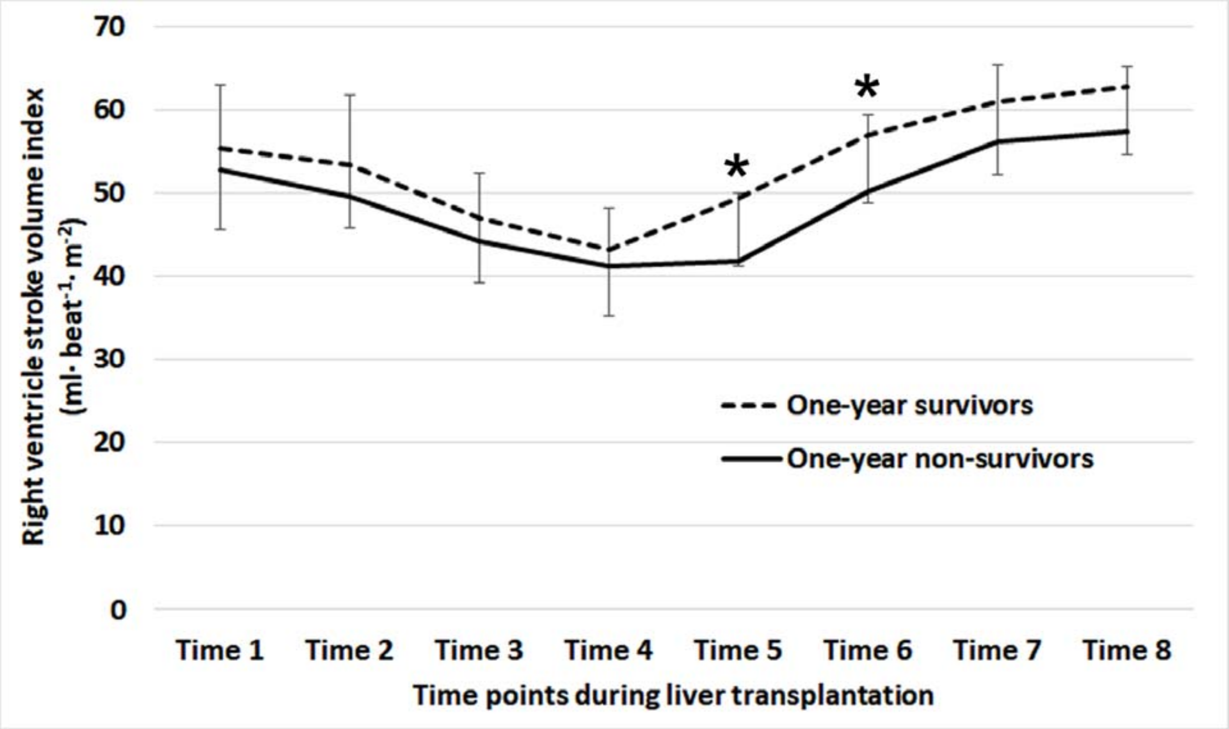

Supplemental Figure S6. Covariate balance plot showing the distribution of standardized differences between the two right ventricular stroke work index groups before and after propensity score matching.

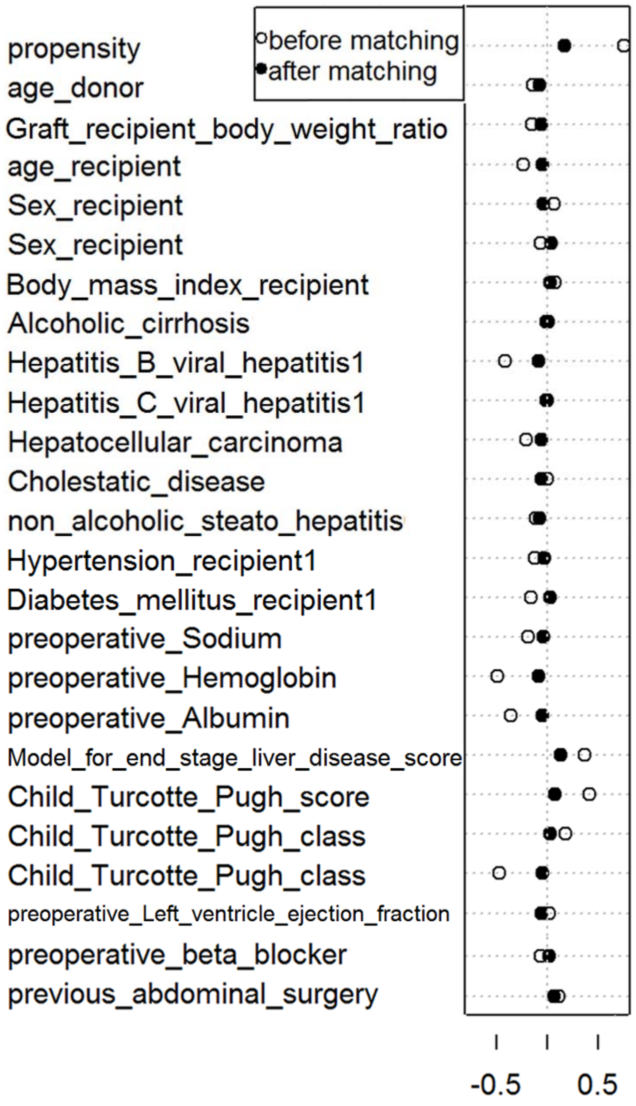

Supplemental Figure S7. Kaplan-Meier survival curve analysis between intraoperative time-weighted mean right ventricular stroke work index groups after propensity score matching. The result of log-rank test between the groups is shown on the figure.

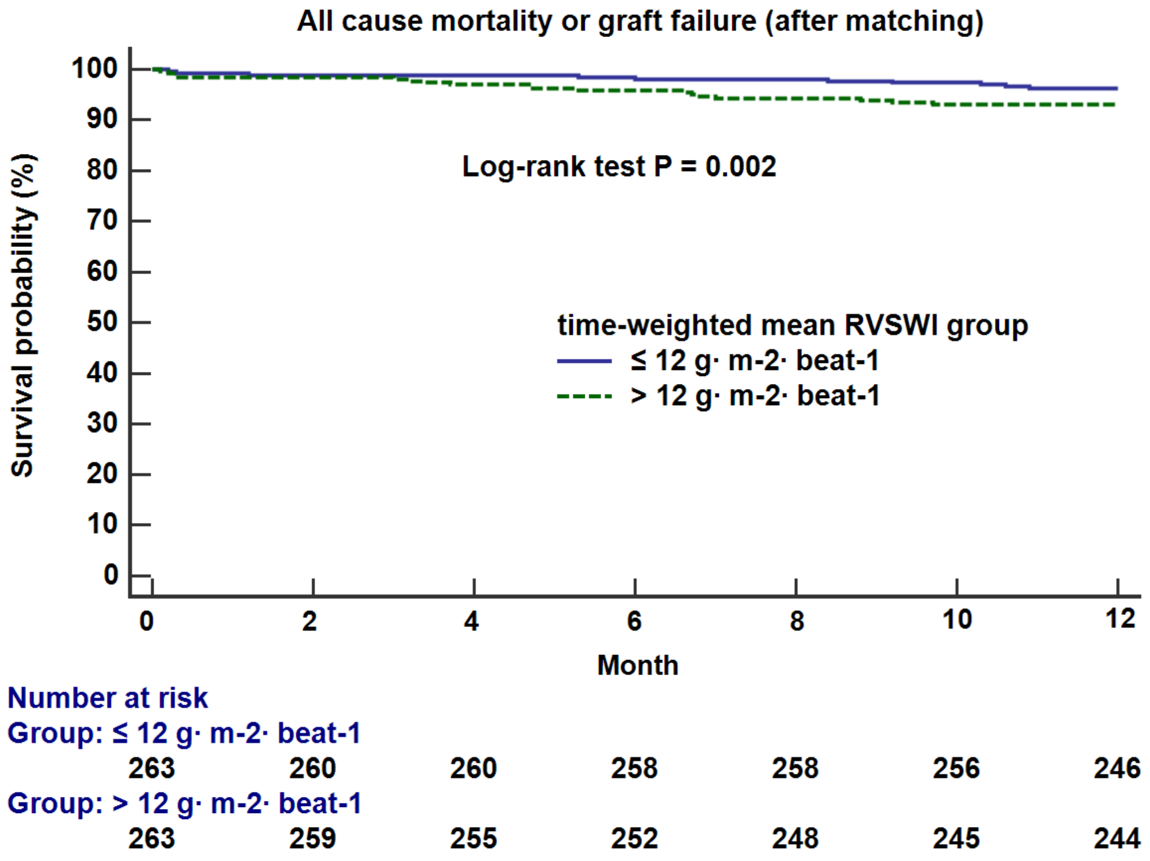

Supplemental Figure S8. Kaplan-Meier survival curve analysis between the four right ventricular stroke work index groups categorized by time-dependent change. The result of log-rank test between the groups is shown on the figure.

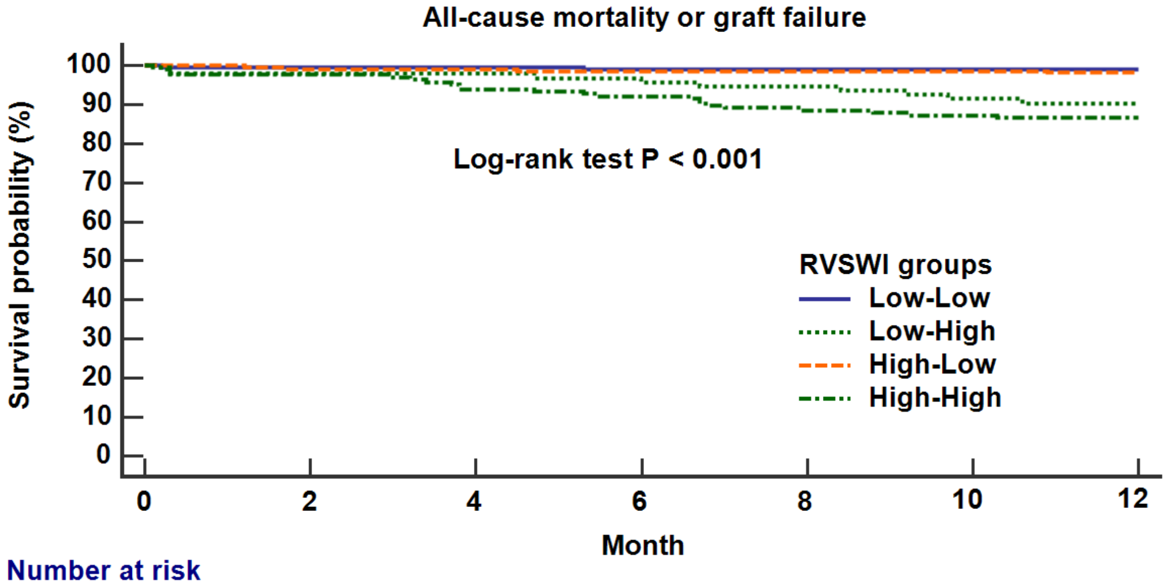

Number at risk

Group	0	2	4	6	8	10	12
Group: Low-Low	213	212	212	211	211	211	206
Group: Low-High	93	91	91	89	88	85	83
Group: High-Low	212	210	210	209	209	209	207
Group: High-High	165	161	155	152	146	144	142

Supplemental Figure S9. Suggested algorithm for hemodynamic management according to right ventricle stroke work index. TEE = transesophageal echocardiography, FiO_2 = fraction of inspired oxygen fraction, PVR = pulmonary vascular resistance, PAOP = pulmonary artery occlusion pressure. We recommend the simultaneous evaluation of right ventricular stroke work index and TEE examination of right heart at the following time-points of liver transplantation: (1) at the end of anesthesia induction as a baseline; (2) at least one time during anhepatic phase; (3) after graft reperfusion; (4) at the end of surgery.

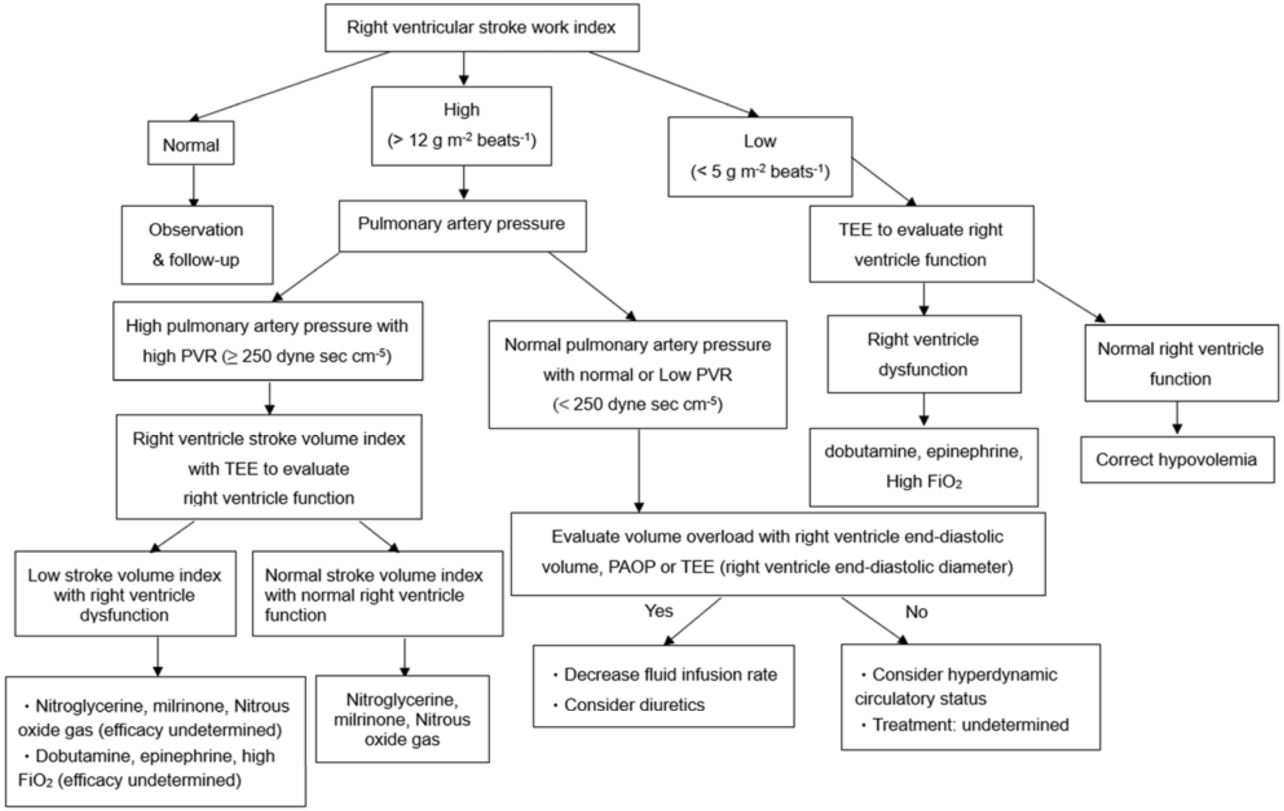

Supplemental Table S1. Comparison of patient characteristics between the patients who were included and excluded from the analysis.

Characteristic	Included patients (n= 683)	Excluded patients (n = 349)	P-value
Demographic data			
Age, years	53 [48, 59]	53 [47, 59]	0.699
Female, n	165 (24.2)	78 (22.3)	0.517
Body-mass index, kg/m^2	23.2 [21.4, 25.3]	23.1 [21.4, 25.4]	0.777
Etiology of liver disease			
Alcoholic liver cirrhosis, n	66 (9.7)	27 (7.7)	0.306
Hepatitis B viral hepatitis, n	266 (38.9)	141 (40.4)	0.651
Hepatitis C viral hepatitis, n	51 (7.5)	29 (8.3)	0.632
Hepatocellular carcinoma, n	371 (54.3)	186 (53.3)	0.755
Cholestatic disease, n	15 (2.2)	10 (2.9)	0.508
Non-alcoholic steatohepatitis, n	38 (5.6)	20 (5.7)	0.912
Baseline medical status			
Hypertension, n	71 (10.4)	34 (9.7)	0.743
Diabetes mellitus, n	82 (12.0)	45 (12.9)	0.681
Preoperative serum sodium, mEq/L	138 [134, 141]	139 [135, 141]	0.379
Preoperative hemoglobin, g/dL	11.1 [9.4, 13.0]	11.2 [9.4, 12.9]	0.943
Preoperative serum albumin level, mg/dL	3.0 [2.6, 3.6]	3.1 [2.6, 3.6]	0.775
Model for end-stage liver disease score	15 [10, 20]	15 [10, 21]	0.613
Child-Turcotte-Pugh score	8 [6, 10]	8 [6, 10]	0.970
Child classification, A/ B/ C, n	187 (27.4)/ 283 (41.4)/ 213 (31.2)	94 (26.9)/ 150 (43.0)/ 105 (30.1)	0.896
Preoperative left ventricle ejection fraction, %	65 [61, 69]	65 [60, 69]	0.732
Preoperative portopulmonary hypertension, n*	10 (1.5)	4 (1.1)	0.676
Preoperative beta-blocker, n	34 (5.0)	16 (4.6)	0.781
Previous abdominal surgery, n	16 (2.3)	6 (1.7)	0.512
Preoperative hepatorenal syndrome, n	-	31 (8.9)	-
With hemodialysis catheter, n	-	318 (91.1)	-
Donor/ graft factors			
Age, years	30 [23, 38]	30 [23, 39]	0.979
Estimated graft-to-recipient weight ratio	1.19 [1.04, 1.40]	1.19 [1.04, 1.40]	0.851

The values are expressed as the median [25th, 75th percentile] or number (%). *Portopulmonary hypertension was diagnosed when mean pulmonary artery pressure >25 mmHg.

Supplemental Table S2. Multivariable Cox proportional hazard regression analysis to predict one-year all-cause mortality or graft failure after liver transplantation after replacing right ventricular stroke work index with mean pulmonary artery pressure and right ventricle stroke index (n=658).

Variable	Hazard ratio (95% CI)	P-value
Age, recipient	1.03 (0.98 – 1.06)	0.241
Female, recipient	0.61 (0.33 – 1.11)	0.121
Alcoholic liver cirrhosis	1.82 (0.79 – 3.85)	0.118
Hepatitis C viral hepatitis	1.99 (0.91 – 4.56)	0.096
Hepatocellular carcinoma	2.01 (1.45 – 3.62)	<0.001
Preoperative hemoglobin, g/dL	0.80 (0.71 – 0.95)	<0.001
Model for end-stage liver disease score	1.11 (0.96 – 1.24)	0.423
Warm ischemic time, min	0.98 (0.96 – 1.02)	0.315
Baseline right ventricle end-diastolic volume, ml	1.02 (1.00 – 1.03)	0.048
Mean SvO_2 during anhepatic phase, %	0.98 (0.94 – 1.03)	0.314
Time-weighted mean of mean pulmonary artery pressure, mmHg	1.04 (1.01 – 1.06)	0.015
Time-weighted mean of right ventricle stroke volume index, $mL \cdot beat \cdot m^{-2}$	0.95 (0.91 – 0.98)	<0.001
Time-weighted mean of left ventricular stroke work index, $g \cdot m^{-2} \cdot beat^{-1}$	1.04 (0.88 – 1.11)	0.271
Intraoperative mean blood glucose, mg/dL	1.02 (0.98 – 1.04)	0.247

CI = confidence interval, Svo_2 = mixed venous oxygen saturation.

Supplemental Table S3. Cause of death during one-year follow-up after transplantation surgery.

**Cause of death**	**Low time-weighted mean right ventricular stroke work index group(≤ 12 g·m^{-2}·beat^{-1}) (n = 317)**	**High time-weighted mean right ventricular stroke work index group (> 12 g·m^{-2}·beat^{-1}) (n = 366)**	**P-value**
Number of death during 5 year	17 (5.4)	48 (13.1)	
Cardiovascular death	2 (0.6)	13 (3.6)	<0.001
Cerebrovascular accident	1 (0.3)	4 (1.1)	
Pulmonary thromboembolism	-	4 (1.1)	
Heart failure	1 (0.3)	5 (1.4)	
Other causes	15 (4.7)	35 (9.6)	0.068
Recurred hepatocellular carcinoma	8 (2.5)	10 (2.7)	
Hepatic failure	3 (2.5)	9 (2.5)	
Hepatitis	2 (0.6)	7 (1.9)	
Liver abscess	-	1 (0.3)	
Other malignancy	1 (0.3)	3 (0.8)	
Renal failure	1 (0.3)	5 (1.4)	

P-values are the results of Fisher's exact test.

Supplemental Table S4. Multivariable logistic regression analysis to predict in-hospital all-cause mortality or graft failure after liver transplantation using time-weighted mean variables (n=658).

Variable	Odds ratio (95% CI)	P-value
Age, recipient	1.06 (1.01 – 1.11)	0.020
Hepatitis B viral hepatitis	0.74 (0.41 – 1.03)	0.169
Hepatocellular carcinoma	1.05 (0.96 – 1.12)	0.195
Model for end-stage liver disease score	1.06 (1.01 – 1.10)	0.010
Cold ischemic time, min	1.02 (1.00 – 1.03)	0.017
Baseline right ventricle end-diastolic volume, ml	1.03 (1.01 – 1.04)	<0.001
Mean Svo_2 during anhepatic phase, %	0.94 (0.90 – 0.98)	0.005
Time-weighted mean of right ventricular stroke work index, $g{\cdot}m^{-2}{\cdot}beat^{-1}$	1.07 (1.04 – 1.12)	<0.001
Time-weighted mean of PVR	1.01 (0.99 – 1.02)	0.058
or PVR at the end of surgery, $dyne{\cdot}sec/cm^5$	1.00 (0.99 – 1.01)	0.178
Intraoperative mean blood glucose, mg/dL	1.01 (1.00 – 1.03)	0.049

CI = confidence interval, Svo_2 = mixed venous oxygen saturation, PVR = pulmonary vascular resistance.

Supplemental Table S5. Multivariable logistic regression analysis to predict in-hospital all-cause mortality or graft failure after liver transplantation using area under curve of the variables (n=658).

Variable	Odds ratio (95% CI)	P-value
Age, recipient	1.05 (0.99 – 1.11)	0.063
Hepatitis B viral hepatitis	0.75 (0.43 – 1.24)	0.124
Hepatocellular carcinoma	1.08 (0.94 – 1.21)	0.186
Model for end-stage liver disease score	1.05 (1.01 – 1.10)	0.018
Cold ischemic time, min	1.02 (1.00 – 1.03)	0.013
Baseline right ventricle end-diastolic volume, ml	1.02 (1.01 – 1.04)	<0.001
Mean Svo_2 during anhepatic phase, %	0.94 (0.90 – 0.99)	0.013
Area under the curve of right ventricular stroke work index ($g{\cdot}m^{-2}{\cdot}beat^{-1}$×24 hour)	1.46 (1.25 – 1.70)	<0.001
Area under the curve of left ventricular stroke work index ($g{\cdot}m^{-2}{\cdot}beat^{-1}$×24 hour)	0.91 (0.80 – 1.03)	0.134
Area under the curve of PVR ($dyne{\cdot}sec/cm^5$×24 hour)	1.02 (0.98 – 1.06)	0.060
Intraoperative mean blood glucose, mg/dL	1.02 (1.00 – 1.03)	0.048

CI = confidence interval, Svo_2 = mixed venous oxygen saturation, PVR = pulmonary vascular resistance.

Supplemental Table S6. Cox proportional hazard regression analysis to predict one-year all-cause mortality or graft failure after liver transplantation using area under curve of the variables (n=658).

Variable	Hazard ratio (95% CI)	P-value
Age, recipient	1.04 (1.01 – 1.07)	0.042
Female, recipient	0.54 (0.31 – 1.09)	0.088
Alcoholic liver cirrhosis	1.91 (0.59 – 3.11)	0.245
Hepatitis C viral hepatitis	1.92 (0.88 – 3.19)	0.084
Hepatocellular carcinoma	1.60 (1.05 – 2.45)	0.045
Preoperative hemoglobin, g/dL	0.81 (0.72 – 0.96)	0.001
Model for end-stage liver disease score	1.11 (1.03 – 1.20)	0.008
Warm ischemic time, min	0.97 (0.94 – 1.00)	0.072
Baseline right ventricle end-diastolic volume, ml	1.01 (0.99 – 1.02)	0.245
Mean SvO_2 during anhepatic phase, %	0.97 (0.96 – 1.03)	0.111
Area under the curve of right ventricular stroke work index ($g{\cdot}m^{-2}{\cdot}beat^{-1}\times24$ hour)	1.24 (1.15 – 1.37)	<0.001
Area under the curve of left ventricular stroke work index ($g{\cdot}m^{-2}{\cdot}beat^{-1}\times24$ hour)	1.01 (0.96 – 1.05)	0.345
Area under the curve of PVR ($dyne{\cdot}sec/cm^5\times24$ hour)	1.01 (0.98 – 1.04)	0.066
Intraoperative mean blood glucose, mg/dL	1.11 (0.97 – 1.02)	0.223

CI = confidence interval, SvO_2 = mixed venous oxygen saturation, PVR = pulmonary vascular resistance.

Supplemental Table S7. Comparison of patient characteristics and perioperative parameters between low and high time-weighted mean right ventricular stroke work index groups before and after propensity score matching.

Characteristic	Before Matching			After Matching		
	Low right ventricular stroke work index group (≤ 12 g·m^{-2}·beat^{-1})	High right ventricular stroke work index group (> 12 g·m^{-2}·beat^{-1})	Standardized difference	Low right ventricular stroke work index group (≤ 12 g·m^{-2}·beat^{-1})	High right ventricular stroke work index group (> 12 g·m^{-2}·beat^{-1})	Standardized difference
Sample size	n = 317	n = 366		n = 263	n = 263	
Demographic data						
Age, years	54 [50, 60]	52 [47, 58]	0.23	54 [49, 60]	52 [48, 58]	0.07
Female, n	72 (22.7)	90 (24.6)	-0.06	69 (26.2)	63 (24.0)	
Body-mass index, kg/m^2	23.1 [21.4, 24.9]	23.3 [21.5, 25.4]	-0.08	22.9 [21.4, 24.9]	23.2 [21.3, 25.2]	-0.08
Etiology of liver disease						
Alcoholic liver cirrhosis, n	30 (9.5)	36 (9.8)	-0.01	26 (9.9)	25 (9.5)	0.01
Hepatitis B viral hepatitis, n	156 (49.2)	110 (30.1)	0.40	107 (40.7)	101 (38.4)	0.05
Hepatitis C viral hepatitis, n	24 (7.6)	27 (7.4)	0.01	21 (8.0)	20 (7.6)	0.01
Hepatocellular carcinoma, n	190 (59.9)	181 (49.5)	0.21	142 (54.0)	136 (51.7)	0.05
Cholestatic disease, n	7 (2.2)	8 (2.2)	0.01	7 (2.7)	4 (1.5)	0.08
Non-alcoholic steatohepatitis, n	13 (4.1)	25 (6.8)	-0.12	13 (4.9)	18 (6.8)	-0.08
Baseline medical status						
Hypertension, n	39 (12.3)	32 (8.7)	0.12	28 (10.6)	25 (9.5)	0.04
Diabetes mellitus, n	46 (14.5)	36 (9.8)	0.14	33 (12.5)	31 (11.8)	0.02
Preoperative serum sodium, mEq/L	139 [135, 141]	138 [132, 140]	0.47	138 (134 – 141)	138 (135 – 141)	0.01
Preoperative hemoglobin, g/dL	11.9 [9.9, 13.5]	10.6 [9.2, 12.3]	0.47	11.3 [9.6, 12.9]	11.1 [9.5, 12.8]	0.06
Preoperative serum albumin level, mg/dL	3.2 [2.7, 3.8]	2.9 [2.5, 3.4]	0.32	3.0 [2.6, 3.5]	3.0 [2.5, 3.5]	0.01
Model for end-stage liver disease score	12 [8, 17]	15 [11, 22]	-0.37	14 [10, 19]	15 [10, 20]	-0.09
Child-Turcotte-Pugh score	8 [5, 10]	8 [7, 11]	-0.42	8 [6, 10]	8 [7, 11]	-0.08
Child class, n (A/ B/ C)	118 (37.2)/ 115 (36.3)/ 84 (26.5)	69 (18.9)/ 168 (45.9)/ 129 (35.2)	-0.36	68 (25.9) / 113 (43.0) / 82 (31.2)	64 (24.3) / 112 (42.6)/ 87 (33.1)	-0.05
Preoperative left ventricle ejection fraction, %	65 [60, 69]	64 [61, 68]	0.07	65 [60, 69]	64 [61, 68]	0.07
Preoperative beta-blocker, n	18 (5.7)	16 (4.4)	0.06	11 (4.2)	12 (4.6)	-0.02
Previous abdominal surgery, n	4 (1.3)	12 (3.3)	-0.14	4 (1.5)	7 (2.7)	-0.08
Donor/ graft factors						
Age, years	30 [24, 39]	30 [23, 37]	0.13	30 [23, 39]	30 [23, 38]	0.07
Estimated graft-to-recipient weight ratio	1.20 [1.04, 1.43]	1.19 [1.04, 1.36]	0.14	1.20 [1.04, 1.41]	1.19 [1.06, 1.38]	0.06

The values are expressed as the median [25th, 75th percentile] or number (%).

Supplemental Table S8. Comparison of postoperative clinical outcomes between the four right ventricular stroke work index groups categorized by time-dependent change.

Variables	Low-low group	Low-high group	High-low group	High-high group	P-value †
Sample size	213 (31.2)	212 (31.0)	93 (13.6)	165 (24.2)	
Length of hospital stay, days	17 [14, 23] ‡	19 [16, 26] §	18 [15, 23] ‡	20 [15, 30]	0.002
Length of intensive care unit stay, days	5 [4, 6] ‡	5 [4, 7]	5 [4, 6]	5 [4, 8]	0.012
Acute kidney injury*, n	62 (29.1) ‡	79 (37.3)	31 (33.3) ‡	86 (52.1)	<0.001
In-hospital mortality, n	3 (1.4) ‡	11 (5.2) ‡	4 (4.3) ‡	27 (16.4)	0.001
One-year mortality, n	7 (3.3) ‡	24 (11.3) §	7 (7.5) ‡	37 (22.4)	0.001

Data are presented as median [25th, 75th percentile] or number (%). *Determined during postoperative seven days and defined by the Kidney Disease Improving Global Outcomes serum creatinine criteria (≥ 1.5 times from baseline).
Groups were determined according to the changes in right ventricular stroke work index between right ventricular stroke work index at T1 and right ventricular stroke work index at T8. For example, low-high group means that right ventricular stroke work index after anesthesia induction (T1) ≤ 12 $g{\cdot}m^{-2}{\cdot}beat^{-1}$ and right ventricular stroke work index at T8 (at the end of surgery) > 12 $g{\cdot}m^{-2}{\cdot}beat^{-1}$. † P-value tests the statistical significance between all groups. ‡ Significantly different from high-high group. § Significantly different from high-low group.

Supplemental Table S9. Baseline echocardiographic findings between two intraoperative time-weighted mean right ventricular stroke work index groups.

Characteristic	Low right ventricular stroke work index group (≤ 12 g·m^{-2}·beat^{-1})	High right ventricular stroke work index group (> 12 g·m^{-2}·beat^{-1})	P-value
Sample size, n	317	366	
Left ventricle ejection fraction, %	65 [60, 69]	65 [61, 68]	0.426
≥ 70%	58 (18.3)	56 (15.3)	0.295
55 – 70%	249 (78.5)	300 (82.0)	0.262
40 – 55%	10 (3.2)	10 (2.7)	0.822
< 40%	-	-	-
Estimated pulmonary artery pressure, mmHg			
Mean	20 [17, 23]	20 [17, 25]	0.086
Systolic pressure	29 [25, 33]	29 [25, 35]	0.069
Degree of tricuspid regurgitation, n			0.752
None or trivial	285 (89.9)	325 (88.8)	
Mild or mild to moderate	27 (8.5)	36 (9.8)	
Moderate or moderate to severe	5 (1.6)	5 (1.4)	
Severe	-	-	
Right atrial size, n			0.536
Collapsed or decreased	2 (0.6)	-	
Normal	286 (90.2)	329 (89.9)	
Mildly dilated	25 (7.9)	32 (8.7)	
Moderately dilated	4 (1.3)	5 (1.4)	
Severely dilated	-	-	

RVSWI = right ventricular stroke work index. Data were presented as median [25th, 75th percentile] or number (%).
